# Synthesis and Antifeedant Activity of Racemic and Optically Active Hydroxy Lactones with the *p*-Menthane System

**DOI:** 10.1371/journal.pone.0131028

**Published:** 2015-07-01

**Authors:** Aleksandra Grudniewska, Marek Kłobucki, Katarzyna Dancewicz, Maryla Szczepanik, Beata Gabryś, Czesław Wawrzeńczyk

**Affiliations:** 1 Department of Chemistry, Wrocław University of Environmental and Life Sciences, Wrocław, Poland; 2 Department of Biology and Ecology, University of Zielona Góra, Zielona Góra, Poland; 3 Department of Invertebrate Zoology, Nicolaus Copernicus University, Toruń, Poland; University of East Anglia, UNITED KINGDOM

## Abstract

Two racemic and two enantiomeric pairs of new δ-hydroxy-γ-lactones based on the *p*-menthane system were prepared from racemic and optically active *cis*- and *trans*-piperitols. The Johnson-Claisen rearrangement of the piperitols, epoxidation of the γδ-unsaturated esters, and acidic lactonization of the epoxy esters were described. The structures of the compounds were confirmed spectroscopically. The antifeedant activities of the hydroxy lactones and racemic piperitone were evaluated against three insect pests: lesser mealworm, *Alphitobius diaperinus* (Panzer); Colorado potato beetle, *Leptinotarsa decemlineata* (Say); and peach-potato aphid, *Myzus persicae* (Sulz.). The chemical transformation of piperitone by the introduction of a lactone moiety and a hydroxy group changed its antifeedant properties. Behavioral bioassays showed that the feeding deterrent activity depended on the insect species and the structure of the compounds. All hydroxy lactones deterred the settling of *M*. *persicae*. Among chewing insects, the highest sensitivity showed *A*. *diaperinus* adults.

## Introduction

*p*-Menthane lactones constitute a family of naturally occurring terpenoid compounds. Several bicyclic γ-lactones of this group, such as mintlactone, isomintlactone, and wine lactone, are well known as flavoring ingredients [1–6]. Synthetic lactones with the *p*-menthane system, in addition to interesting odoriferous attributes [7–9], exhibit valuable biological activities such as antifungal [10] and antifeedant properties [11–16].

Our interest in terpenoid lactones is inspired mainly by their activity as insect feeding deterrents; many natural antifeedants contain the lactone moiety and have isoprenoid structures [17–20]. However, their low concentrations in plants and their typically complex syntheses have limited the large-scale application of natural antifeedants. Therefore, in our opinion, synthetic feeding deterrents with simple structures offer better potential for practical use in insect pest population control. Thus, we have synthesized a number of terpenoid lactones by using optically active monoterpenoids as the starting materials [13–15, 21, 22].

In many syntheses, we obtained the final products as enantiomeric pairs. Our studies confirmed that the biological effects of the optically active substances depend on their configuration. Moreover, structural modifications of natural monoterpenoids, such as lactonization, iodolactonization, and incorporation of hydroxyl and carbonyl groups, can cause changes in their biological activities. Some of the prepared compounds were very effective antifeedants against selected insect pests. Their activities are comparable with that of the most active known antifeedant, azadirachtin [11, 15, 16].

We studied three insect pests: the lesser mealworm, *Alphitobius diaperinus* (Panzer) is a common cosmopolitan insect pest of poultry houses, particularly in broiler sheds. Its mass occurrence in poultry farms creates serious veterinary and economic problems for poultry breeders: the beetles damage the insulation of the houses and have the potential to act as reservoirs for poultry parasites and pathogens [23, 24]. The beetle is also known to be a pest in animal feeds, especially in neglected storage rooms.

The Colorado potato beetle, *Leptinotarsa decemlineata* (Say), is widely regarded as the most important insect defoliator of plants in the family Solanaceae in North America, Europe, and Asia. High selection pressure, together with a natural propensity to adapt to toxic substances, has finally resulted in a large number of insecticide-resistant *L*. *decemlineata* populations. The beetle has developed resistance to insecticides from different chemical groups [25, 26]. Consequently, alternative methods for the control of this pest are needed.

Aphids, in addition to their direct detrimental effects on host plants due to the uptake of phloem sap, are the most important vectors of plant viruses. They transmit nearly 30% of all hitherto described plant virus species, which account for nearly 50% of all insect-borne viruses [27]. Therefore, it is crucial to deter aphid probing and feeding at pre-ingestional, ingestional, and post-ingestional phases. In our former study, we found that the chlorinated, brominated, and iodinated lactone derivatives of piperitone deterred the probing, feeding, and settling of the peach-potato aphid *Myzus persicae* (Sulz.), in contrast to piperitone itself, which appeared to be a weak attractant [13].

Here, we present the stereoselective synthesis of racemic and enantiomeric pairs of new δ-hydroxy-γ-lactones (**6a-c**, **11a-c**) with the *p*-menthane system. The lactones were obtained from racemic and enantiomerically enriched (ee = 91–98%) *cis*- (**1a-c**) and *trans*-piperitols (**7a-c**), which were synthesized from racemic piperitone as described earlier [28]. The optically active and racemic hydroxy lactones, as well as piperitone, were examined for antifeedant activity against *A*. *diaperinus*, *L*. *decemlineata*, and *M*. *persicae*.

## Materials and Methods

### General

Analytical TLC was performed on Merck Kieselgel 60 F_254_ plates with mixtures of hexane and diethyl ether in various ratios. Compounds were detected by spraying the plates with 1% Ce(SO_4_)_2_ and 2% H_3_[P(Mo_3_O_10_)_4_] in 10% H_2_SO_4_, followed by heating to 120–200°C. Column chromatography was performed on silica gel (Kieselgel 60, 230–400 mesh ASTM, Merck) with mixtures of hexane and diethyl ether (in various ratios) as eluents.

Gas chromatography (GC) analysis was carried out on a 6890N GC instrument equipped with a flame ionization detector (FID) using H_2_ as the carrier gas and a capillary column (Trace TR-5, 30 m × 0.32 mm × 1.0 μm). Chiral gas chromatography was carried out on a CP-Chirasil-DEX CB (25 m × 0.25 mm × 0.25 μm) column. Enantiomeric excesses were determined with the following temperature programs: for **6a**-**b**, 120°C, 170°C (1°C/min), 200°C (20°C/min) (9.5 min), total run time = 61.00 min, *t*_R_ **6b** = 42.54 min, *t*_R_ **6a** = 43.50 min; for **11a**-**b**, 120°C, 180°C (1°C/min), 200°C (20°C/min) (10 min), total run time = 71.00 min, *t*_R_ **11a** = 48.00 min, *t*_R_ **11b** = 50.45 min. ^1^H NMR, ^13^C NMR, DEPT 135, ^1^H–^1^H COSY, and HSQC spectra were recorded in CDCl_3_ solution on a Bruker Avance DRX 300 MHz or BrukerAvance II 600 MHz spectrometer. Chemical shifts were referenced to the residual solvent signal (δ_H_ 7.26, δ_C_ 77.0). IR spectra were recorded for the liquid films or as KBr plates on a Thermo-Nicolet IR300 FT-IR spectrometer. Optical rotations were determined on an Autopol IV automatic polarimeter in chloroform solution with concentrations denoted in g/100 mL. CH elemental analyses were performed on an EA-1110 elemental analyzer.

### Reagents

*m*-Chloroperbenzoic acid was purchased from Sigma-Aldrich (Poznań, Poland). Racemic and optically active γ,δ-unsaturated esters (**2a**-**c** and **8a**-**c**) were synthesized from the corresponding *cis*- and *trans-*piperitols as described earlier [13].

### Chemical synthesis

#### Synthesis of epoxy esters (3a-c, 4a-c, and 9a-c)

All epoxy esters were obtained according to the following general procedure. A solution of *m*-chloroperbenzoic acid (3 mmol) in dry methylene chloride (15 mL) was added dropwise to a stirred solution of γ,δ-unsaturated ester (3 mmol) in methylene chloride (20 mL). The mixture was stirred at room temperature for 24 h. Then the reaction mixture was washed with saturated Na_2_S_2_O_3_ solution, and extracted with methylene chloride. The separated organic layer was washed with 0.5 M NaHCO_3_ solution, dried over anhydrous MgSO_4_, and concentrated *in vacuo*. The crude product (or mixture of products) was purified by column chromatography (hexane/diethyl ether, gradient from 9:1 to 4:1). The yields of the reactions and physical and spectral data for the epoxy esters obtained are given below.

**Ethyl (±)-(*t*-2’,t-3’-epoxy-*c*-4’-isopropyl-1’-methylcyclohex-*r*-1’-yl)acetate** [(±)**-3c**] and **ethyl (±)-(*c*-2’,*c*-3’-epoxy-c-4’-isopropyl-1’-methylcyclohex-*r*-1’-yl)acetate** [(±)-**4c**]: The mixture of racemic epoxy esters (±)-**3c** and (±)-**4c** (53 and 47%, respectively, according to GC, light yellow liquid, 0.46 g, yield 75%) was obtained from racemic ester (±)-**2c** (0.56 g, 2.50 mmol): ^1^H NMR (300 MHz; CDCl_3_) δ_H_ 0.95 (d, *J* = 6.8 Hz, 6H, (CH_3_)_2_CH− of **3c**), 0.98 and 1.02 (two d, *J* = 6.8 Hz, 6H, (CH_3_)_2_CH− of **4c**), 1.16 (s, 3H, CH_3_-1’ of **4c**), 1.17 (s, 3H, CH_3_-1’ of **3c**), 1.25 and 1.26 (two t, *J* = 7.1 Hz, 6H, −OCH_2_CH_3_), 1.29–1.34 (m, 4H), 1.45–1.54 (m, 2H), 1.67 (m, 1H), 1.73 (m, 1H), 2.21 and 2.57 (two d, *J* = 14.6 Hz, 2H, CH_2_-2 of **4c**), 2.34 (s, 2H, CH_2_-2 of **3c**), 2.94 and 3.00 (two d, *J* = 3.8 Hz, 2H, H-2’ and H-3’ of **4c**), 2.96 (dd, *J* = 3.7 and 1.1 Hz, 1H, H-3’ of **3c**), 3.24 (dd, *J* = 3.7 and 1.9 Hz, 1H, H-2’ of **3c**), 4.13 and 4.14 (two q, *J* = 7.1Hz, 4H, −OCH_2_CH_3_).

**Ethyl (1’*S*,2’*S*,3’*R*,4’*R*)-(2’,3’-epoxy-4’-isopropyl-1’-methylcyclohex-1’-yl)acetate** (**3a**) and **ethyl (1’*S*,2’*R*,3’*S*,4’*R*)-(2’,3’-epoxy-4’-isopropyl-1’-methylcyclohex-1’-yl)acetate** (**4a**): The mixture of epoxy esters **3a** and **4a** (53 and 47%, respectively, according to GC, light yellow liquid, 0.21 g, yield 72%) was obtained from ester (–)-**2a** (0.27 g, 1.20 mmol, ee = 98%). The ^1^H NMR spectrum of this mixture was identical to that of the mixture of racemic epoxy esters (±)-**3c** and (±)-**4c**.

**Ethyl (1’*R*,2’*R*,3’*S*,4’*S*)-(2’,3’-epoxy-4’-isopropyl-1’-methylcyclohex-1’-yl)acetate** (**3b**) and **ethyl (1’*R*,2’*S*,3’*R*,4’*S*)-(2’,3’-epoxy-4’-isopropyl-1’-methylcyclohex-1’-yl)acetate** (**4b**): The mixture of epoxy esters **3b** and **4b** (53 and 47%, respectively, according to GC, light yellow liquid, 0.89 g, yield 72%) was obtained from ester (+)-**2b** (1.15 g, 5.13 mmol, ee = 91%). The ^1^H NMR spectrum of this mixture was identical to that of the mixture of racemic epoxy esters (±)-**3c** and (±)-**4c**.

**Ethyl (±)-(*c*-2’,*c*-3’-epoxy-t-4’-isopropyl-1’-methylcyclohex-*r*-1’-yl)acetate** [(±)-**9c**]: Racemic epoxy ester (±)-**9c** (light yellow liquid, 0.87 g, yield 90%) was obtained from racemic ester (±)-**8c** (0.90 g, 4.02 mmol): ^1^H NMR (300 MHz; CDCl_3_) δ_H_ 0.94 and 0.97 (two d, *J* = 6.8 Hz, 6H, (CH_3_)_2_CH−), 1.12 (s, 3H, CH_3_-1’), 1.17 (m, 1H), 1.25 (t, *J* = 7.1 Hz, 3H, −OCH_2_CH_3_), 1.30–1.40 (m, 2H), 1.64 (m, 1H), 1.74 (m, 1H), 2.31 and 2.44 (two d, *J* = 13.8 Hz, 2H, CH_2_-2), 2.94 and 2.98 (two d, *J* = 3.8 Hz, 2H, H-2’ and H-3’), 4.12 and 4.13 (two q, *J* = 7.1Hz, 2H, −OCH_2_CH_3_).

**Ethyl (1’*S*,2’*R*,3’*S*,4’*S*)-(2’,3’-epoxy-4’-isopropyl-1’-methylcyclohex-1’-yl)acetate** (**9a**): Epoxy ester **9a** (light yellow liquid, 1.26 g, yield 95%) was obtained from ester (+)-**8a** (1.24 g, 5.54 mmol, ee = 94%). Its ^1^H NMR spectrum was identical to that of (±)-**9c**.

**Ethyl (1’*R*,2’*S*,3’*R*,4’*R*)-(2’,3’-epoxy-4’-isopropyl-1’-methylcyclohex-1’-yl)acetate** (**9b**): Epoxy ester **9b** (light yellow liquid, 1.53 g, yield 89%) was obtained from ester (−)-**8b** (1.60 g, 7.14 mmol, ee = 98%). Its ^1^H NMR spectrum was identical to that of (±)-**9c**.

#### Synthesis of δ-hydroxy-γ-lactones (6a-c and 11a-c)

All δ-hydroxy-γ-lactones were obtained according to the following general procedure. To the solution of epoxy ester (or mixture of epoxy esters, 2 mmol) in THF (10 mL) were added water (5 mL), and 5 drops of HClO_4_ (70%). The mixture was stirred at room temperature for 24 h, and extracted with diethyl ether. The separated ethereal solution was washed with saturated NaHCO_3_ solution, brine, dried over anhydrous MgSO_4_, and concentrated *in vacuo*. The crude product was purified by column chromatography (hexane/diethyl ether, 4:1). The yields of the reactions and physical and spectral data of the hydroxy lactones obtained are given below.

**(±)-*t*-5-Hydroxy-*c*-4-isopropyl-*r*-1-methyl-7-oxa-*cis*-bicyclo[4.3.0]nonan-8-one** [(±)-**6c**]: Racemic hydroxy lactone (±)-**6c** (amorphous crystals, mp 54–56°C, 0.28 g, yield 80%) was obtained from the mixture (0.40 g, 1.67 mmol) of racemic epoxy esters (±)-**3c** (53%) and (±)-**4c** (47%): ^1^H NMR (600 MHz; CDCl_3_) δ_H_ 0.80 and 0.94 (two d, *J* = 7.0 Hz, 6H, (CH_3_)_2_CH−), 1.10 (m, 1H, one CH_2_-3), 1.18 (s, 1H, CH_3_-1), 1.27 (m, 1H, H-4), 1.39 (ddd, *J* = 14.5, 12.8 and 4.4 Hz, 1H, H-2, axial), 1.58 (m, 1H, one CH_2_-3), 1.77 (s, 1H, −OH), 1.82 (dt, *J* = 14.5 and 3.3 Hz, 1H, H-2, equatorial), 2.03 and 2.53 (two d, *J* = 17.1 Hz, 2H, CH_2_-9), 2.20 (septet d, *J* = 7.0 and 2.8 Hz, 1H, (CH_3_)_2_CH−), 3.37 (ddd, *J* = 10.3, 7.9, and 1.5 Hz, 1H, H-5), 3.91 (d, *J* = 7.9 Hz, 1H, H-6); ^13^C NMR δ_C_ (151 MHz; CDCl_3_) 15.72 and 20.77 (CH_3_)_2_CH−), 19.02 (C-3), 25.22 (CH_3_)_2_CH−), 28.57 (CH_3_-1), 32.93 (C-2), 38.95 (C-9), 40.33 (C-1), 45.34 (C-4), 74.05 (C-5), 92.28 (C-6), 176.63 (C-8); IR (KBr, ν_max_/cm^−1^) 3372 (b, m), 2957 (s), 1778 (s), 1462 (m), 1159 (m). Anal. Calcd for C_12_H_20_O_3_ (212.29): C, 67.89; H, 9.50. Found: C, 65.80; H, 9.57.

**(+)-(1*S*,4*R*,5*R*,6*R*)-5-Hydroxy-4-isopropyl-1-methyl-7-oxabicyclo[4.3.0]-nonan-8-one** [(+)-**6a**]: Hydroxy lactone (+)-**6a** (colorless oily liquid, 0.14 g, yield 89%, ee = 98%) was obtained from the mixture (0.18 g, 0.75 mmol) of epoxy esters **3a** (53%) and **4a** (47%). [α]_D_^29^ = +21.8° (*c* 2.15 in CHCl_3_). Its IR and NMR spectra were identical to those of (±)-**6c**.

**(−)-(1*R*,4*S*,5*S*,6*S*)-5-Hydroxy-4-isopropyl-1-methyl-7-oxabicyclo[4.3.0]-nonan-8-one** [(−)-**6b**]: Hydroxy lactone (−)-**6b** (colorless oily liquid, 0.42 g, yield 72%, ee = 91%) was obtained from the mixture (0.66 g, 2.75 mmol) of epoxy esters **3b** (53%) and **4b** (47%). [α]_D_^29^ = –20.1° (*c* 4.17 in CHCl_3_). Its IR and NMR spectra were identical to those of (±)-**6c**.

**(±)-*t*-5-Hydroxy-*t*-4-isopropyl-*r*-1-methyl-7-oxa-*cis*-bicyclo[4.3.0]nonan-8-one** [(±)-**11c**]: Racemic hydroxy lactone (±)-**11c** (colorless oily liquid, 0.27 g, yield 77%) was obtained from racemic epoxy ester (±)-**9c** (0.39 g, 1.62 mmol): ^1^H NMR (600 MHz; CDCl_3_) δ_H_ 0.96 and 0.97 (two d, *J* = 6.7 Hz, 6H, (CH_3_)_2_CH−), 1.16 (m, 1H, H-4), 1.28 (s, 3H, CH_3_-1), 1.36–1.55 (m, 3H, CH_2_-3 and one CH_2_-2), 1.57–1.63 (m, 2H, one CH_2_-2 and (CH_3_)_2_CH−), 1.66 (s, 1H, −OH), 2.26 and 2.41 (two d, *J* = 16.7 Hz, 2H, CH_2_-9, AB system), 4.12 (d, *J* = 2.8 Hz, 1H, H-6), 4.29 (m, 1H, H-5); ^13^C NMR (151 MHz; CDCl_3_) δ_C_ 18.81 (C-3), 20.31 and 21.11 (CH_3_)_2_CH−), 22.37 (CH_3_-1), 28. 17 (CH_3_)_2_CH−), 33.69 (C-2), 37.54 (C-1), 42.72 (C-4), 46.03 (C-9), 66.94 (C-5), 85.92 (C-6), 176.05(C-8); IR (film, ν_max_/cm^−1^) 3478 (b, s), 2956 (s), 1772 (s), 1460 (m), 1190 (m), 1016 (s). Anal. Calcd for C_12_H_20_O_3_ (212.29): C, 67.89; H, 9.50. Found: C, 65.50; H, 9.52.

**(−)-(1*S*,4*S*,5*R*,6*R*)-5-Hydroxy-4-isopropyl-1-methyl-7-oxabicyclo[4.3.0]-nonan-8-one** [(−)-**11a**]: Hydroxy lactone (−)-**11a** (colorless oily liquid, 0.78 g, yield 80%, ee = 94%) was obtained from epoxy ester **9a** (1.10 g, 4.58 mmol). [α]_D_^28^ = –23.5° (*c* 3.0 in CHCl_3_). Its IR and NMR spectra were identical to those of (±)-**11c**.

**(+)-(1*R*,4*R*,5*S*,6*S*)-5-Hydroxy-4-isopropyl-1-methyl-7-oxabicyclo[4.3.0]-nonan-8-one [(+)-11b**]: Hydroxy lactone (+)-**11b** (colorless oily liquid, 0.54 g, yield 92%, ee = 98%) was obtained from epoxy ester **9b** (0.66 g, 2.75 mmol). [α]_D_^27^ = +26.6° (*c* 3.0 in CHCl_3_). Its IR and NMR spectra were identical to those of (±)-**11c**.

### Feeding deterrent activity tests

Experiments were conducted using *A*. *diaperinus*, *L*. *decemlineata*, and *M*. *persicae*. Choice and no-choice tests were used according to the procedures described previously [13, 29, 30]. Detailed information about feeding deterrent activity tests and insect cultures is provided in the S1 File [13, 29–35].

#### Chewing insects (*A*. *diaperinus* and *L*. *decemlineata*)

From the test data for chewing insects (*A*. *diaperinus* and *L*. *decemlineata*), the deterrence coefficients (relative *R*, and absolute *A*) were calculated using Eqs (1) and (2) [36, 37]:

$$R=\frac{\left(C-E\right)}{\left(C+E\right)}x100$$ (1)

$$A=\frac{\left(CC-EE\right)}{\left(CC+EE\right)}x100$$ (2)

where *C* and *CC* are the weights of the control consumed by the insects in the choice and no-choice tests, respectively, and similarly, *E* and *EE* are the weights of the treated food consumed, respectively.

The measure of the deterrent activity of the tested compounds is the total coefficient of deterrence, Eq (3):

T=A+R (3)

The total coefficient of deterrence, *T*, which ranged from −200 to 200, serves as an index of activity. Compounds with *T* ranging from 151 to 200 are classified as very strong deterrents, and those with values from 101 to150 and from 51 to 100 are classified as good deterrents and compounds with medium activity, respectively. Compounds with *T* values lower than 50 are poor antifeedants. Negative *T* values indicate attraction. In practice, the deterrent activity of the chemical compounds is significant in a no-choice situation.

To estimate and compare larval and adult feeding levels in the no-choice tests, the amount of treated food consumed was expressed as a percentage of the consumption in the control according to Eq (4):

$$\frac{EE}{CC}x100$$ (4)

The mean values of the deterrence coefficients were compared using one-way analysis of variance (ANOVA) followed by Tukey’s test at a significance level of *p* < 0.05 [38]. The mean values of consumption by the larvae and adults in the no-choice test were compared using Student’s *t*-test.

#### Aphids (*M*. *persicae*)

In the experiment with *M*. *persicae*, the settling choice-test was applied as described previously [13]. From the test data, the relative index of deterrence (*DI*) was calculated using Eq (5):

$$DI=\frac{\left(C-T\right)}{\left(C+T\right)}$$ (5)

where *C* is the number of aphids that settled on the control leaf and *T* is the number of aphids that settled on the leaf treated using the studied compound. The values of *DI* range between +1 (ideal deterrent) and −1 (ideal attractant). The test data were analyzed using the Student’s *t*-test.

Aphid probing and, especially, the phloem sap uptake by *M*. *persicae* was monitored in the no-choice situation by using the electrical penetration graph (EPG) technique, i.e., the electronic registration of aphid stylet penetration in plant tissues, according to the procedure described previously [13, 39]. The parameters derived from the EPGs, related to activities in the peripheral and vascular tissues, were analyzed according to their frequency and duration. The results were statistically analyzed using the Mann-Whitney *U* test at *p* < 0.05.

## Results and Discussion

### Synthesis of hydroxy lactones

**Fig 1:**
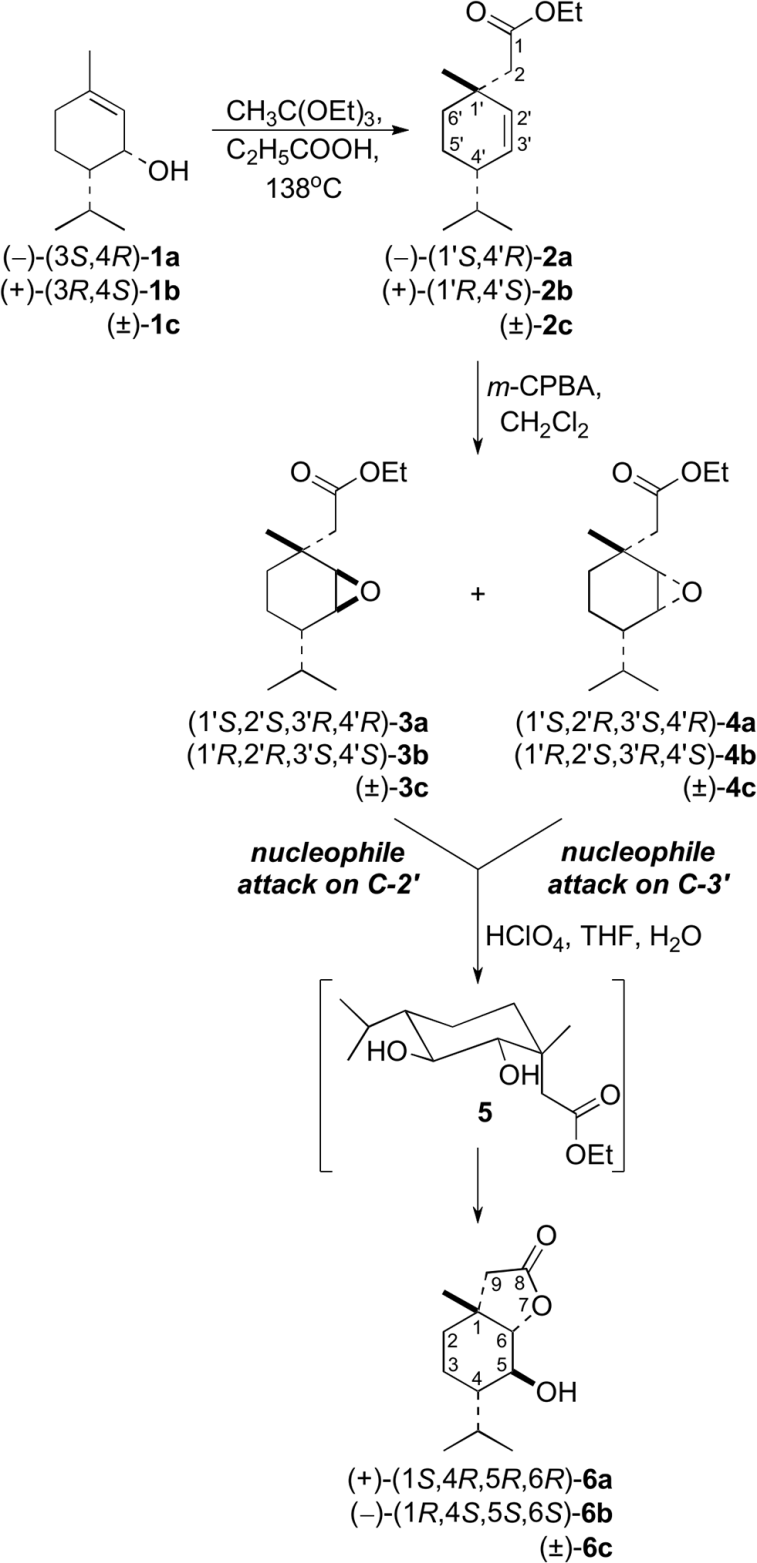
Synthesis of hydroxy lactones 6a-c.

**Fig 2:**
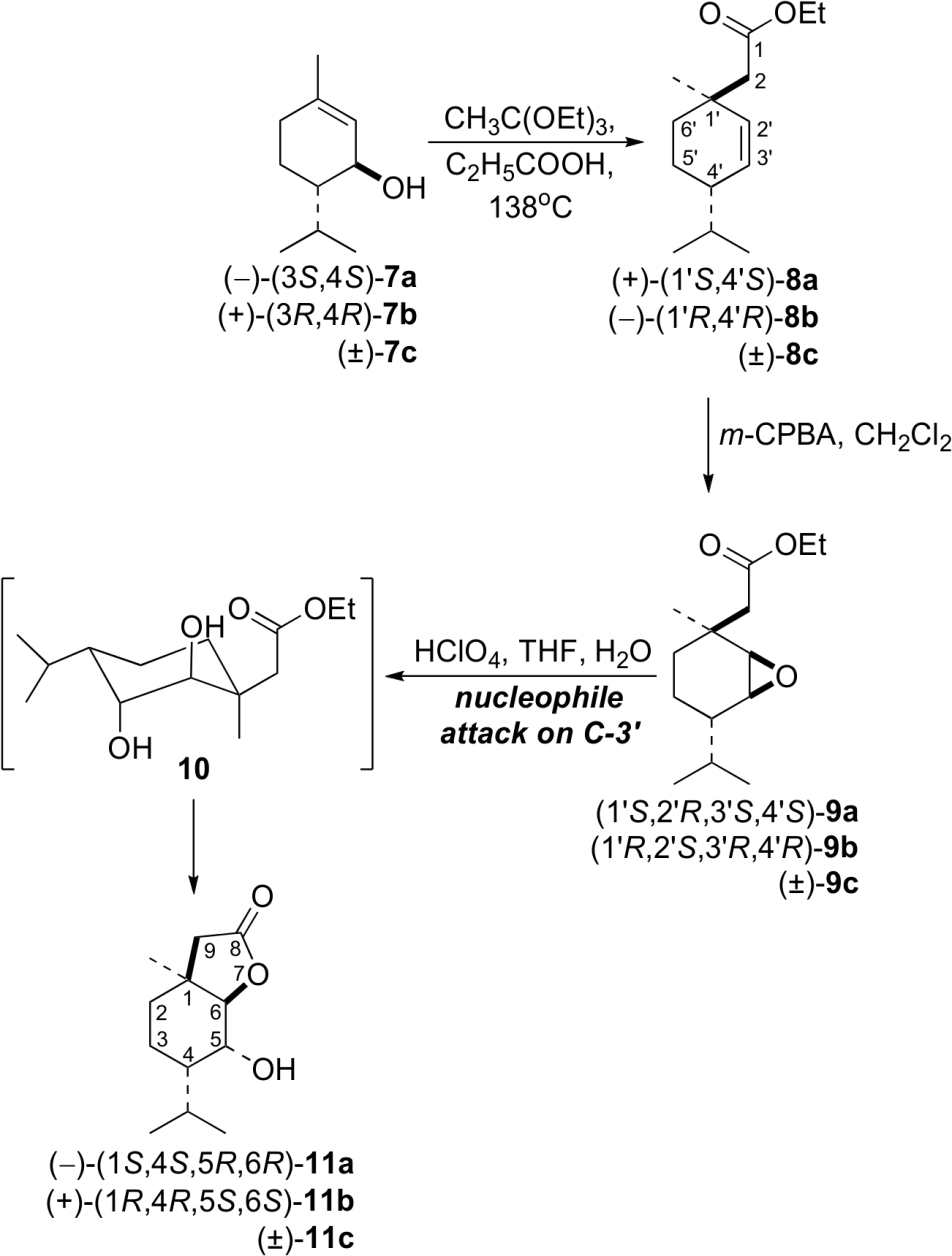
Synthesis of hydroxy lactones 11a-c.

Two racemic and two enantiomerically enriched pairs of new δ-hydroxy-γ-lactones, **6a**-**c** and **11a**-**c**, with the *p*-menthane system were synthesized from *cis*- and *trans*-piperitols, **1a**-**c** (Fig 1) and **7a**-**c** (Fig 2), respectively, which were prepared from (±)-piperitone [28]. Enantiomerically enriched *cis*- (**1a**,**b**) and *trans*-piperitols (**7a**,**b**) were obtained from racemic piperitols (**1c** and **7c**, respectively) *via* their enzymatic esterification with vinyl propionate in the presence of lipase Amano PS [28]. Racemic and optically active γ,δ-unsaturated esters (**2a**-**c** and **8a**-**c**) were obtained *via* the Johnson-Claisen- rearrangement of the corresponding allylic alcohols, as described earlier [13].

The epoxidation of *cis*-esters (**2a**-**c**) with *m*-chloroperbenzoic acid afforded a mixture of epoxy esters **3a**-**c** and **4a**-**c**, respectively (Fig 1). GC analysis of these mixtures indicated, that one epoxy ester was formed in a slight diastereoisomeric excess (53:47%). Unfortunately, these mixtures were inseparable by column chromatography. The structures of the products were confirmed by ^1^H NMR spectroscopy. According to analysis by Dreiding models, the doublets of doublets at 3.24 and 2.96 ppm were ascribed to H-2’ and H-3’, respectively, of the *trans*-isomer of epoxy ester (**3c**), and the broad doublets at 3.00 and 2.94 ppm to these protons (H-2’ and H-3’, respectively) in the *cis*-isomer (**4c**). Protons H-2’ and H-3’ in the *trans*-isomer (**3c**) are more deshielded (in comparison to *cis*-isomer, **4c**) because they are located on the same side of the cyclohexane ring as the carboethoxymethylene group. From the integration of these signals, it follows that the mixture contains 53% **3c** and 47% **4c**. Two doublets at 2.21 and 2.57 ppm (*J* = 14.6 Hz) were ascribed to the methylene protons (CH_2_-2) of the carboethoxymethylene group of the *cis*-isomer of epoxide (**4c**), and the two-proton singlet at 2.34 ppm to these protons in the *trans*-isomer (**3c**). In epoxy ester **4c,** the isopropyl group is oriented *cis* to the oxirane ring. Taking this into consideration, the two doublets (*J* = 6.8 Hz) at 0.98 and 1.02 ppm were ascribed to the methyl protons of the isopropyl group of diastereoisomer **4c**. The six-proton doublet at 0.95 ppm (*J* = 6.8 Hz) was ascribed to the isopropyl methyl protons of epoxy ester **3c**.

The key step of the synthesis was the acidic lactonization of the obtained epoxy esters. The reaction of mixtures of **3a** and **4a**, **3b** and **4b**, and **3c** and **4c**, catalyzed by HClO_4_ in THF/H_2_O solution, gave only the single corresponding δ-hydroxy-γ-lactone (**6a**, **6b**, and **6c**, respectively, Fig 1). The structures of these products were confirmed by their spectral data. In the IR spectrum of lactone **6c**, the characteristic absorption bands of the γ-lactone moiety (1778 cm^−1^) and the hydroxy group (3372 cm^−1^) were observed. The presence of a doublet (δ = 3.91) for H-6 coupled with neighboring proton H-5 (*J* = 7.9 Hz) in the ^1^H NMR spectrum of **6c** proved their *trans*-diaxial orientation, and consequently, the equatorial positions of the C−OH as well as the lactone C−O bonds. Additionally, the values of the coupling constants (*J*_H5-H4_ = 10.3 Hz, *J*_H5-H6_ = 7.9 Hz, and *J*_H5-H3e_ = 1.5 Hz) found for the doublet of doublets of doublets at 3.37 ppm in H-5 indicates the axial orientation of H-4, and therefore, the *trans*-diequatorial positions of the isopropyl and hydroxy groups.

The formation of only a single hydroxy lactone from both isomers of the epoxy esters can be explained on the basis of the mechanism suggested for lactonization of another epoxy ester with the *p*-menthane framework [22]. The product of the proposed first step, the opening of the epoxide to the diol ester, was not observed in the reaction mixture, because it most likely cyclized immediately to the γ-lactone. The diol results from the nucleophilic attack of H_2_O on C-2’ or C-3’ from the opposite side of the oxonium ion which is formed after H^+^ addition to the oxirane oxygen. In the case of the *trans*-epoxides (**3a**-**c**), attack at C-2’ leads to *trans*-diequatorial diols **5** with the carboethoxymethylene group in the axial position (Fig 1). The same dihydroxy esters (**5**) can be obtained in the case of nucleophile attack on C-3’ in the *cis*-epoxides (**4a**-**c**). These diols (**5**) undergo lactonization to the corresponding δ-hydroxy-γ-lactones (**6a**-**c**). The enantiomeric excesses of hydroxy lactones **6a** and **6b**, determined by chiral GC, were the same as the starting γ,δ-unsaturated esters (**2a** and **2b**): 98% and 91%, respectively (S7 Fig).

Oxidation of γ,δ-unsaturated esters **8a**-**c** with *m*-chloroperbenzoic acid gave only one epoxy ester (**9a**-**c**, respectively, Fig 2). The comparison of the ^1^H NMR spectra of **9c** and the mixture of epoxides **3c** and **4c** allows the determination of the configuration (*cis* or *trans*) of the oxirane ring relative to the carboethoxymethylene group. Two broad doublets (*J* = 3.7 Hz) at 2.93 and 2.98 ppm for H-2’ and H-3’, respectively, in the ^1^H NMR spectrum of **9c** were observed. The shapes and chemical shifts of these signals are analogous to the multiplets for H-2’ and H-3’ in epoxide **4c**. Additionally, two doublets (δ = 2.31 and 2.44, *J* = 13.8 Hz) from the methylene protons (CH_2_-2) of the carboethoxymethylene group, similarly to **4c**, were observed. These data indicate that, in epoxy ester **9c**, the oxirane ring is oriented *cis* to the carboethoxymethylene group. In the same manner as in the synthesis of racemic epoxy ester **9c**, the oxidation of the optically active γ,δ-unsaturated esters (**8a** and **8b**) afforded the corresponding epoxides (**9a** and **9b**, respectively).

The acidic lactonization of epoxy esters **9a**-**c** gave δ-hydroxy-γ-lactones **11a**-**c**, respectively. The presence of the hydroxy group and the γ-lactone ring in lactone **11c** was confirmed by the absorption bands in the IR spectrum at 3478 and 1772 cm^−1^, respectively. In the ^1^H NMR spectrum of **11c**, multiplets due to H-5 (4.29 ppm) and a doublet due to H-6 (4.12 ppm) were present. The small value of the coupling constant, *J*_H5-H6_ = 2.8 Hz, proved the *trans*-diequatorial orientation of the coupled protons. Therefore, it can be concluded that the C−OH as well as the lactone C−O bonds are located in axial positions. The enantiomeric excesses of lactones **11a** (ee = 94%) and **11b** (ee = 98%), determined by chiral GC (S8 Fig), were identical to the enantiomeric excesses of the γ,δ-unsaturated esters (**8a** and **8b**, respectively) which were used for the reaction.

Similarly to the synthesis of lactones **6a**-**c**, we assume that the lactonization of epoxy esters **9a**-**c** proceeds *via* the diols (**10**, Fig 2). In the case of epoxides **9a**-**c**, nucleophilic attack at C-3’ leads to *trans*-diaxial diols **10** with the carboethoxymethylene group in the equatorial position.

### Biological activities

#### Lesser Mealworm, *Alphitobius diaperinus*

The antifeedant activity results for piperitone and the hydroxy lactones (**6a**-**c** and **11a**-**c**) toward *A*. *diaperinus* are presented in Table 1.

**Table 1:** Feeding deterrent activity of piperitone and the hydroxy lactones (6a-c and 11a-c) against *Alphitobius diaperinus* (Panzer).

Compound	Deterrence coefficients					
	Larvae			Adults		
	A	R	T	A	R	T
**Piperitone**	−4.76 ab	25.07 a	20.31 a	12.24 a	69.21 b	81.45 a
**6a**	11.98 ab	43.51 a	54.49 ab	92.58 c	20.73 a	111.23 a
**6b**	31.54 b	86.18 b	117.72 b	76.85 bc	72.38 b	149.23 ab
**6c**	20.15 ab	26.15 a	46.30 ab	51.23 ab	67.62 b	118.85 a
**11a**	−19.53 a	13.68 a	−5.85 a	93.61 c	78.28 b	171.89 b
**11b**	4.47 ab	48.14 a	52.61 ab	77.58 bc	61.44 b	139.02 ab
**11c**	14.20 ab	31.82 a	46.02 ab	94.09 c	86.20 b	180.29 b

Values represent the means of four replicates, each set by using ten larvae or adults (n = 40). *A*, absolute coefficient (no-choice test); *R*, relative coefficient (choice test); *T*, total coefficient (*T = A + R*). Means followed by the same letters within each column are not significantly different (one-way ANOVA and Tukey’s tests, *p* < 0.05).

**Fig 3:**
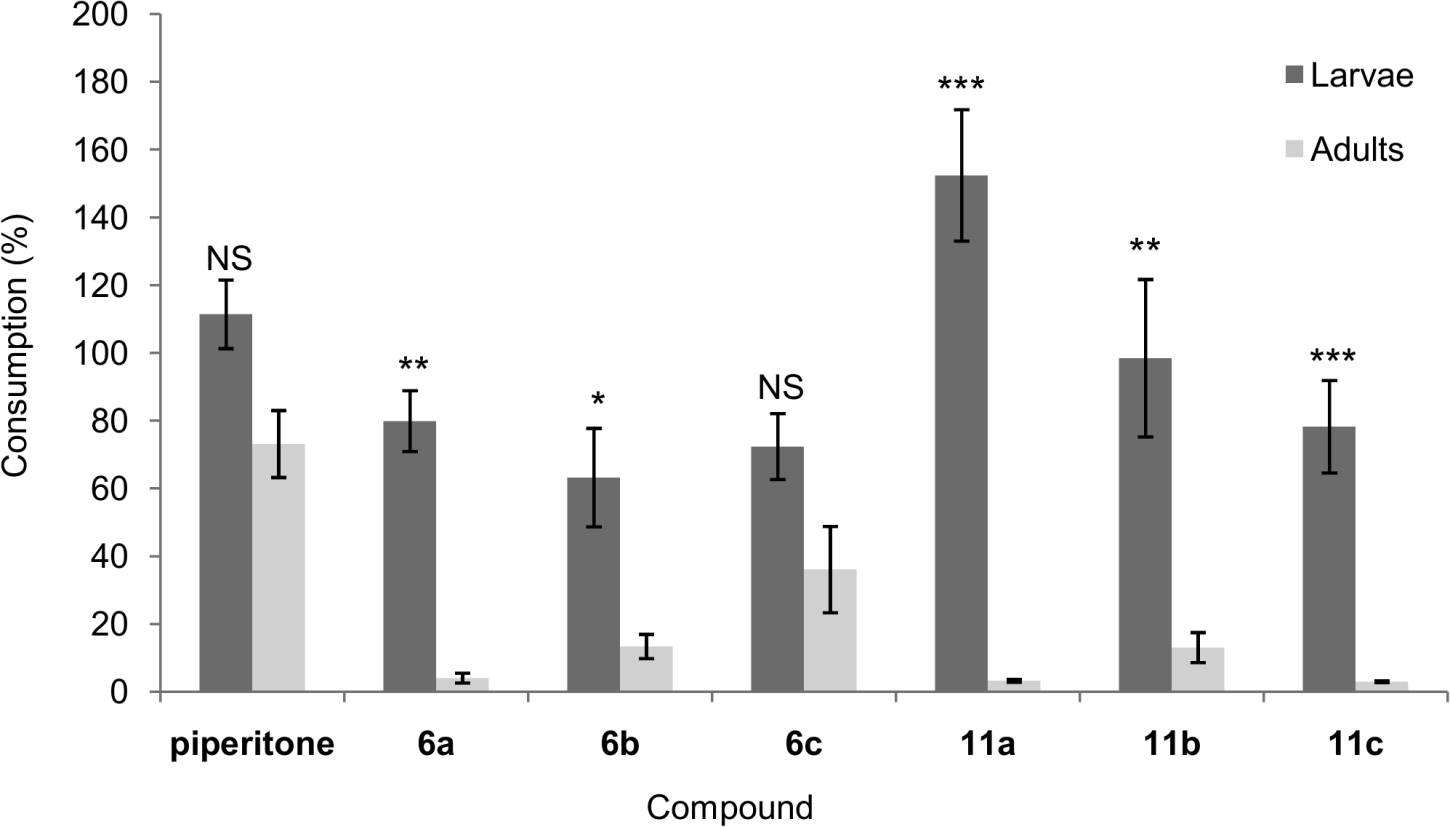
Effects of piperitone and hydroxy lactones (6a-c and 11a-c) on the feeding of *Alphitobius diaperinus* larvae and adults in no-choice tests. Data are expressed as percentages of control consumption. Values represent the means of four replicates, each set by using ten larvae or adults (n = 40). The standard error is indicated on each bar. * *p* < 0.05; ** *p* < 0.01; *** *p <* 0.001; NS: not significant (Student’s *t*-test).

The starting piperitone showed weak deterrent activity for both developmental stages, especially in the no-choice test. What is more, in the trials using larvae, piperitone stimulated higher food intake compared to that by the control, and therefore, it is defined as an attractant. Likewise, its lactone derivative **11a** showed attractant properties. The consumption of food treated with this compound (**11a**) was the highest (152.43% compared to the control) (Fig 3). Among the tested hydroxy lactones, only **6b** was a very good antifeedant against *A*. *diaperinus* larvae, but only in the choice test. In the no-choice situation, its activity was moderate. Larvae feeding decreased by approximately 40% compared to the control (Fig 3). The remaining compounds were weak antifeedants.

The sensory sensitivity of *A*. *diaperinus* adults was significantly higher compared with the larvae. All the hydroxy lactones were better antifeedants with rescpect to piperitone. The most active compounds were **11a** and **11c**, with activities comparable to that of the most active known antifeedant, azadirachtin [30]. In the presence of these lactones (**11a** and **11c**), the food consumed by the adults in the no-choice test represented only around 3% of the consumption in the control (Fig 3). Isomer **11b**, with the 1*R*,4*R*,5*S*,6*S* configuration, was a weaker antifeedant than its enantiomer **11a** or racemic lactone **11c**. High activity in the no-choice test was observed for hydroxy lactone **6a**. The low value of the relative coefficient *R* for this compound was because of the low level of feeding in the control. This may suggest that lactone **6a** operates as a repellent. In this case, a strong reduction in treated food consumption in both tests and only low discrimination between the treated and control foods in the choice test was observed [40]. Thus, the change in configuration from 1*S*,4*R*,5*R*,6*R* (**6a**) to 1*S*,4*S*,5*R*,6*R* (**11a**) leads to change in properties and model of operation. The same situation was observed in the case of the pair of lactones, **6a** and **6b**. The coefficients *A* and *R* obtained for hydroxy lactones **6b** and **11a** clearly indicate their antifeedant properties, with a somewhat weaker activity for isomer **6b**. The activity of **6c** in terms of the model of operation was similar to that of lactone **6b**. Despite the fact that compounds **6a** and **6c** had very similar *T*-values, their activities and action models were different. Hydroxy lactones **6b** and **11b** showed similar activities (consumption in relation to the control was about 13% in the no-choice tests) (Fig 3). In the choice test, the activity of isomer **6b** was slightly higher, but without significant difference.

#### Colorado Potato Beetle, *Leptinotarsa decemlineata*

**Fig 4:**
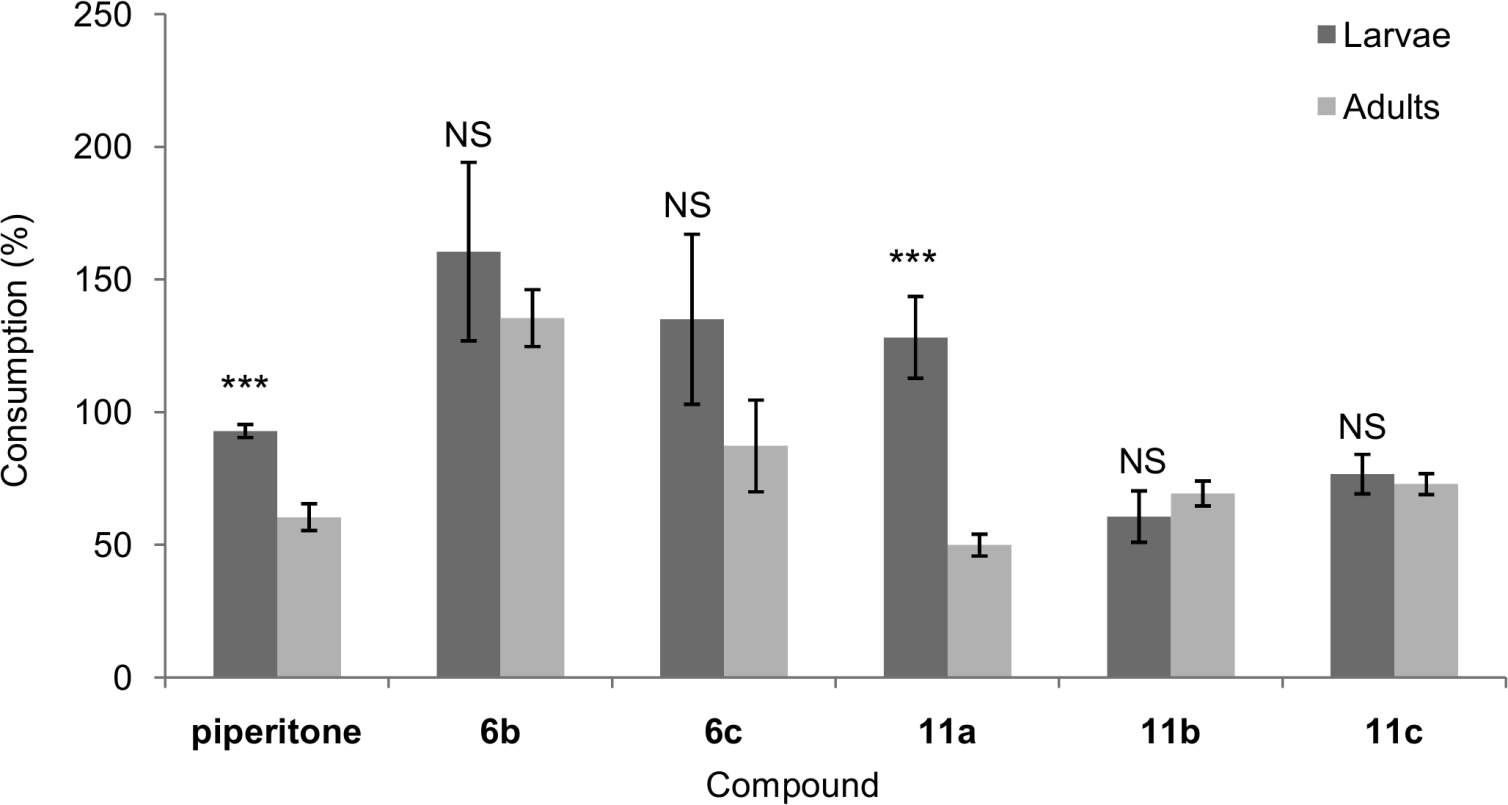
Effects of piperitone and hydroxy lactones (6b,c and 11a-c) on the feeding of *Leptinotarsa decemlineata* larvae and adults in no-choice tests. Data are expressed as percentages of control consumption. Values represent the means of four replicates, each set by using ten larvae or adults (n = 40). The standard error is indicated on each bar. *** *p* < 0.001; NS: not significant (Student’s *t*-test).

The tested compounds demonstrated weak antifeedant activity against *L*. *decemlineata* (Table 2). Piperitone did not reduce the feeding behavior, especially of larvae in the no-choice test. No significant difference was noted between area consumed of the piperitone-treated leaf disks and untreated ones. Disks treated with hydroxy lactones **6b**, **6c**, and **11a** in the no-choice tests were eaten at a higher proportion than the control disks. The disk treated with lactone **6b** were the most willingly consumed (consumption in relation to the control was 160.6%) (Fig 4).

**Table 2:** Feeding deterrent activity of piperitone and hydroxy lactones (6a-c and 11a-c) against *Leptinotarsa decemlineata* (Say).

Compound	Deterrence coefficients					
	Larvae			Adults		
	A	R	T	A	R	T
**Piperitone**	3.43 abc	21.9 ab	25.33 ab	24.97 bc	6.38 a	31.35 abc
**6a**	nt^a^	nt	nt	nt	nt	nt
**6b**	−18.50 a	8.42 a	−10.08 a	−11.21 a	17.52 ab	6.31 a
**6c**	−10.82 ab	29.61 ab	18.79 ab	12.89 ab	12.36 a	25.25 ab
**11a**	−11.29 ab	55.88 bc	44.59 b	33.63 c	41.96 bc	75.59 d
**11b**	25.58 c	69.53 c	95.11 c	18.34 bc	54.78 c	73.12 d
**11c**	12.62 bc	43.47 b	56.09 b	15.81 bc	41.32 bc	57.13 bcd

Values represent the means of four replicates, each set by using ten larvae or adults (n = 40). *A*, absolute coefficient (no-choice test); *R*, relative coefficient (choice test); *T*, total coefficient (*T = A + R*). Means followed by the same letters within each column are not significantly different (one-way ANOVA and Tukey’s tests, *p* < 0.05).

^a^Not tested.

The change in configuration of the chiral centers from 1*R*,4*S*,5*S*,6*S* (**6b**) to 1*S*,4*S*,5*R*,6*R* (**11a**) only slightly decreased the value of the coefficient *A*, and these compounds remained attractants in the no-choice test (Table 2). In this study, *L*. *decemlineata* larvae were deterred only by hydroxylactone **11b** during the choice tests, where they consumed significantly more control disks than the treated ones. However, in the no-choice bioassays, this compound (**11b**) reduced herbivory of the treated leaf disks by approximately 40% (Fig 4).

The test compounds also showed low antifeedant activity against *L*. *decemlineata* adults. Thus, although comparison of the total deterrence coefficients revealed the strongest activities for the hydroxy lactones **11a** and **11b,** from a practical point of view, they are rather weak deterrents (Table 2).

#### Aphids, *Myzus persicae*

The relationship between aphids and plants is in some ways unique among insects. Aphids feed on phloem sap using their specialized sucking-piercing mouthparts, which penetrate plant tissues intercellularly until they reach the vascular tissue [41]. Plant penetration can be divided into three distinct phases: the pathway, xylem, and phloem phases. During pathway, the insects’ stylets (aphid mouthparts) pierce through the epidermis and non-vascular tissues; the xylem phase involves the uptake of xylem sap; and the phloem phase comprises the main feeding, but is always preceded by sieve element salivation, presumably to suppress phloem wound responses [42]. Of the three probing phases, the pathway and phloem phases are crucial steps in the chemosensory-based host-plant selection and host-plant acceptance processes. The parameters derived from the EPG describe aphid behavior during probing and feeding and are good indicators of plant suitability or the interference of probing by chemical or physical factors, including exogenously applied chemicals, in individual plant tissues [43]. Accordingly, the alteration of aphid behavior during the pathway phase may reflect the hindrance of probing at a pre-ingestional level; changes in behavior during contact with the phloem elements may denote an ingestional effect; and the refusal to settle on plants even if the feeding process has not been impeded may be a symptom of post-ingestional deterrence [13, 14, 44].

**Fig 5:**
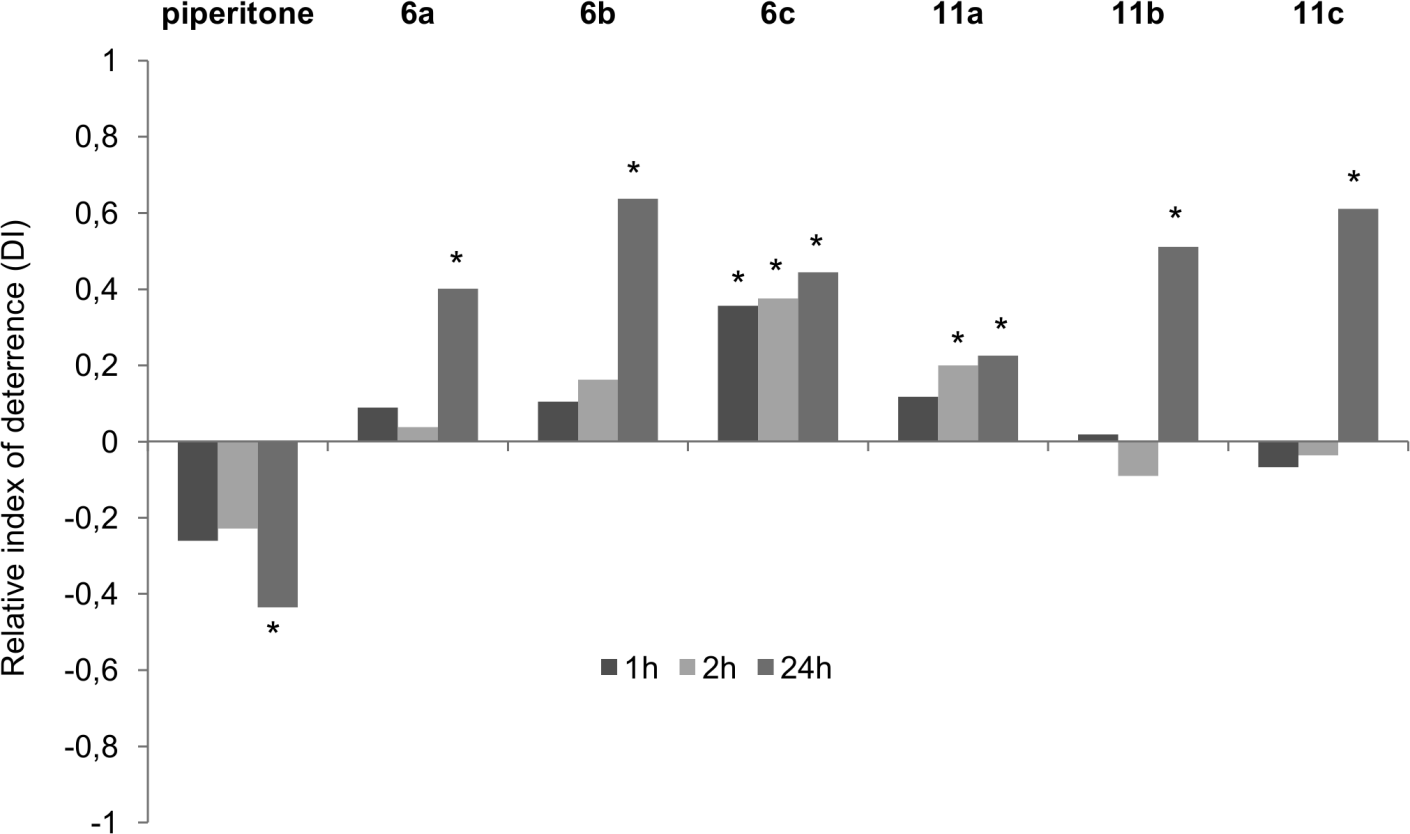
Relative index of deterrence (*DI*) of piperitone and hydroxy lactones (6a-c and 11a-c) 1, 2, and 24 h after application. Asterisks (_*_) indicate statistically significant biological activity according to the Student's *t*-test results (*p* < 0.05); *DI* < 0, attractant; *DI* > 0, deterrent (n = 8 replicates, 20 viviparous apterous females/replicate).

All hydroxy lactones (**6a-c** and **11a-c**) evoked negative responses in the *M*. *persicae*, and the intensity of the effect increased over time. However, the deterrent activity of the individual compounds varied in potency, time of expression, and duration of the effect, depending on the spatial structure of the lactones and biological background of their activity (Fig 5).

Hydroxy lactones **6a**-**c** and **11a**-**c** deterred the settling of *M*. *persicae*, in contrast to piperitone, which showed weak attractant properties (*DI*_24_ = −0.43) (Fig 5). The lactone **6c** prevented aphid settling as soon as 1 h after application, and the index of deterrence remained at a relatively high level until the end of the 24 h experiment (*DI*_1_ = 0.36, *DI*_2_ = 0.38, and *DI*_24_ = 0.44). The deterrent effect of the remaining hydroxy lactones occurred later than 2 h after application. The relative indices of deterrence *DI*_24_ of these lactones ranged from 0.23 (**11a**) to 0.64 and 0.61 (**6b** and **11c**, respectively) (Fig 5).

The monitoring of aphid probing behavior *via* EPG revealed that lactone **6b** was an active deterrent at the pre-ingestional and ingestional levels (Table 3, S9–S11 Figs, S2 File). The pre-ingestional activity was manifested during the pathway phase, that is, during aphid probing in non-phloem tissues. A reduction of the average probing time and increase in the number of short probes that did not reach beyond the epidermis occurred, and fewer aphids reached phloem vessels after the application of **6b**. The ingestional activity was demonstrated in the considerable reduction of sap ingestion activities in both total time and individual ingestion periods and the failure to sustain the ingestion after sampling phloem sap in a number of individuals. The activity of lactone **11c** was expressed after the ingestion of phloem sap. There was no significant decrease in the ingestion time, either total or individual. However, the considerably higher number of epidermal and mesophyll probes following ingestion periods may indicate negative changes in the phloem sap quality due to the application of **11c**. None of the studied behavioral aspects of aphid probing was affected after the application of lactones **11a** and **11b**. Nevertheless, the avoidance of **11a**- and **11b**-treated leaves by aphids was found 24 h after application. This may suggest post-ingestional effects of these lactones, probably due to metabolic reasons.

**Table 3:** Effects of piperitone and hydroxy lactones (6b and 11a-c) on probing activities of *Myzus persicae* (Sulz.).

EPG parameter	Control	Piperitone	6b	11a	11b	11c
**General aspects of aphid probing behavior** ^a^						
Total duration of nonprobing (min)	23.1 ± 8.1^b^	35.9 ± 8.4^b^ a	29.9 ± 5.1^c^	28.3 ± 7.7^b^	32.4 ± 8.5^b^	68.8 ± 15.4^b^ a
Total duration of pathway (min)	193.6 ± 29.7^b^	232.0 ± 22.7^b^	337.9 ± 29.5^c^ ab	203.0 ± 36.2^b^	280.9 ± 35.0^b^	257.1 ± 37.0^b^
Total duration of the phloem phase (min)	214.2 ± 45.9^b^	192.2 ± 30.7^b^	80.4 ± 32.0^c^ b	241.9 ± 39.9^b^	144.7 ± 37.1^b^	119.3 ± 37.1^b^
Total duration of the phloem sap ingestion phase (min)	212.0 ± 45.8^b^	189.5 ± 30.6^b^	78.1 ± 31.9^c^ b	231.0 ± 38.8^b^	142.5 ± 37.2^b^	116.6 ± 37.2^b^
Total duration of derailed stylet activities (min)	0.0^b^	5.7 ± 5.7^b^	5.9 ± 5.9^c^	0.0^b^	0.0^b^	8.8 ± 8.8^b^
Duration of xylem sap uptake (min)	49.1 ± 29.8^b^	14.1 ± 9.1^b^	25.9 ± 13.1^c^	6.8 ± 2.9^b^	22.1 ± 12.5^b^	26.1 ± 15.6^b^
Proportion of the phloem phase in total probing (%)	46.3 ± 9.6^b^	42.7 ± 6.7^b^	17.7 ± 6.9^c^ b	53.0 ± 8.4^b^	31.7 ± 8.0^b^	28.3 ± 8.1^b^
Mean duration of nonprobing (min)	3.0 ± 0.9^b^	2.1 ± 0.4^b^	± 0.1^c^ ab	2.3 ± 0.4^b^	± 0.4^b^	1.0 ± 0.6^b^
No. of probes	6.8 ± 1.6^b^	19.1 ± 3.9^b^ a	27.6 ± 5.5^c^ a	12.3 ± 2.4^b^	15.9 ± 4.1^b^	22.0 ± 4.0^b^ a
No. of short probes (pathway < 3 min)	1.3 ± 0.6^b^	7.3 ± 1.8^b^ a	10.9 ± 3.8^c^ a	4.0 ± 1.1^b^	7.1 ± 2.5^b^	9.3 (±2.6) ^b^ a
Duration of first probe (min)	94.1 ± 52.3^b^	7.3 ±5.1^b^ a	30.6 ± 10.9^c^ b	33.7 ± 13.0^b^	69.4 ± 40.1^b^ b	66.2 ± 40.4^b^ b
Mean duration of a probe (min)	144.6 ± 46.5^b^	34.2 ± 5.7^b^ a	19.7 ± 3.3^c^ a	59.9 ± 12.6^b^	100.6 ± 39.8^b^	62.0 ± 38.3^b^ a
**General aspects of aphid behavior related to probing in phloem elements** ^d^						
Proportion of salivation in the total phloem phase (%)	12.1 ± 8.9^e^	2.9 ± 1.5^b^	16.3 ± 10.4^f^	5.7 ± 2.6^b^	4.3 ± 2.1^g^	18.8 ± 9.9^e^
No. of probes to the first phloem phase	6.0 ± 1.4^e^	12.3 ± 3.3^b^	12.8 ± 4.1^f^	6.6 ± 1.4^b^	7.9 ± 1.7^g^	12.2 ± 4.0^e^
Duration of the shortest pathway wave before the phloem phase (min)	36.6 ± 6.1^e^	33.6 ± 5.5^b^	15.2 ± 3.3^f^ a	20.9 ± 5.7^b^	27.6 ± 7.7^g^	41.7 ± 15.0^e^
Duration of nonprobe period before the first phloem phase (min)	22.1 ± 8.7^e^	26.0 ± 8.2^b^	15.6 ± 5.7^f^	18.7 ± 7.4^b^	14.0 ± 4.2^g^	34.1 ± 14.9^e^
Time from the first probe to first phloem phase (min)	160.4 ± 34.2^e^	167.1 ± 34.8^b^	195.7 ± 71.2^f^	154.0 ± 34.2^b^	148.9 ± 35.2^g^	186.2 ± 46.5^e^
Time from the beginning of probing to the first phloem phase (min)	43.8 ± 5.6^e^	40.6 ± 6.1^b^	18.6 ± 4.5^f^ ab	26.8 ± 5.6^b^	30.0 ± 7.5^g^	45.2 ± 14.4^e^
Duration of the first phloem phase (min)	152.7 ± 55.4^e^	27.3 ± 11.8^b^	53.5 ± 27.9^f^	154.8 ± 43.6^b^	102.1 ± 42.5^g^	77.9 ± 40.2^e^
No. of probes after the first phloem phase	1.0 ± 0.5^e^	6.8 ± 3.5^b^	14.5 ± 8.7^f^ a	5.7 ± 1.8^b^	10.8 ± 4.7^g^	11.0 ± 3.1^e^ a
No. of probes (< 3 min) after the first phloem phase	0.1 ± 0.1^e^	2.6 ± 1.3^b^	6.8 ± 5.1^f^ a	2.1 ± 0.8^b^	6.2 ± 2.9^g^	5.0 ± 1.8^e^ a
**Aphid behavior related to phloem sap ingestion** ^d^						
No. of phloem sap ingestion phases	1.0 ± 0.3^e^	4.6 ± 0.8^b^	2.7 ± 0.8^f^	2.8 ± 0.5^b^	3.6 ± 0.6^g^	3.7 ± 0.8^g^
Mean duration of phloem sap ingestion phase (min)	173.5 ± 51.2^e^	56.8 ± 12.8^b^	34.3 ± 13.6^f^	131.0 ± 37.1^b^	73.5 ± 32.2^g^	81.1 ± 43.8^g^
Time from the first probe to first phloem sap ingestion phase (min)	161.0 ± 34.2^e^	168.0 ± 34.7^b^	211.6 ± 76.4^f^	172.4 ± 33.3^b^	149.5 ± 35.2^g^	159.0 ± 39.5^g^
Time from the beginning of probing to the first phloem sap ingestion phase (min)	44.4 ± 5.7^e^	41.4 ± 6.1^b^	21.9 ± 3.9^f^	36.7 ± 7.8^b^	30.7 ± 7.4^g^	48.1 ± 15.8^g^
No. of sustained phloem sap ingestion phases (> 10 min)	1.5 ± 0.3^g^	2.4 ± 0.3^e^	2.3 ± 0.5^h^	2.3 ± 0.3^e^	2.1 ± 0.4^i^	2.6 ± 0.5^i^
Time from the first probe to first sustained phloem sap ingestion phase (10 min) (min)	206.8 ± 37.3^g^	157.7 ± 31.9^e^	114.2 ± 69.8^h^	155.9 ± 31.5^e^	209.0 ± 44.6^i^	158.0 ± 37.2^i^
Time from the beginning of that probe to first sustained phloem sap ingestion phase (10 min) (min)	43.2 ± 4.7^g^	52.2 ± 7.7^e^	21.1 ±5.4^h^ a	39.3 ± 8.1^e^	40.2 ± 9.5^i^	51.3 ± 17.2^i^

Values represent the mean ± standard error; ‘a’ and ‘b’ represent statistically significant differences in relation to control and piperitone, respectively (*p* < 0.05, Mann-Whitney *U* test).

^a^All aphids were included in the analysis; if an aphid did not show a phloem phase, the value of the phloem-related parameter was shown as zero.

^b^n = 12.

^c^n = 7.

^d^Only aphids that showed a phloem phase (at least E1), phloem sap ingestion phase (E2), or sustained phloem sap ingestion phase (E2 > 10 min), respectively, were included in the analysis.

^e^n = 11.

^f^n = 6.

^g^n = 10.

^h^n = 4.

^i^n = 9.

## Conclusions

Racemic (**6c**, **11c**) and enantiomerically enriched pairs of δ-hydroxy-γ-lactones (**6a**,**b** and **11a**,**b**) with the *p*-menthane system were obtained *via* stereoselective synthesis from *cis*- (**1a**-**c**) and *trans*-piperitols (**7a**-**c**).

The incorporation of the γ-lactone moiety and hydroxy group into the piperitone molecule changed its biological activity. Behavioral bioassays showed that the antifeedant activity of the studied lactones was related to the insect species and structure of the compounds. Among the chewing insects, *A*. *diaperinus* adults showed the highest sensitivity, with the hydroxy lactones, except for **6c**, strongly limiting their feeding. All the piperitone derivatives were much stronger antifeedants for adults than for larvae, which is a quite commonly noted phenomenon. This is because the adults possess a larger number of mouthpart chemoreceptors and choose specific host plants and sites for oviposition [45].

All the hydroxy lactones deterred the settling of *M*. *persicae*; this was in contrast to piperitone, which showed weak attractant properties. Lactone **6b** was the strongest deterrent at the pre-ingestional and ingestional levels. Among the piperitone derivatives, no clear effect was noted related to the configuration of the chiral centers on the deterrent activity of the tested compounds.

## Supporting Information

S1 Fig ^1^H NMR spectrum of mixture of 3c and 4c. CDCl_3_, 300 MHz.
(TIF)

S2 Fig ^1^H NMR spectrum of 9c. CDCl_3_, 300 MHz.
(TIF)

S3 Fig ^1^H NMR spectrum of 6c. CDCl_3_, 600 MHz.
(TIF)

S4 Fig ^13^C NMR spectrum of 6c. CDCl_3_, 151 MHz.
(TIF)

S5 Fig ^1^H NMR spectrum of 11c. CDCl_3_, 600 MHz.
(TIF)

S6 Fig ^13^C NMR spectrum of 11c. CDCl_3_, 151 MHz.
(TIF)

S7 Fig CGC chromatograms of 6a-c. (a) (±)-**6c**; (b) (+)-**6a**, ee = 98%; (c) (−)-**6b**, ee = 91%.
(TIF)

S8 Fig CGC chromatograms of 11a-c. (a) (±)-**11c**; (b) (−)-**11a**, ee = 94%; (c) (+)-**11b**, ee = 98%.
(TIF)

S9 Fig Analysis of aphid probing activities on plants after application of piperitone and lactones 6b and 11a-c, expressed as the proportion of behavioral events in individual aphids. Numbers on the *x-*axis represent individual aphids. np, no probing; C, probing in parenchymatous tissues; E1, salivation in phloem; E2, ingestion of phloem sap; F, derailed stylet activities; G, ingestion of xylem sap (n = 12).
(TIF)

S10 Fig Sequential changes in aphid probing behavior over 8 h post-application of piperitone and lactones 6b and 11a-c (n = 12). (TIF)

S11 Fig Cumulative proportion of aphids that showed probing activities in sieve elements, phloem salivation (E1), and sap ingestion (E2) over 8 h post-application of piperitone and lactones 6b and 11a-c (n = 12). (TIF)

S1 File Bioassays. Insect culture and feeding deterrent activity tests.
(DOCX)

S2 File Aphid probing. (DOCX)

## References

[1] Takahashi K, Someya T, Muraki S, Yoshida T. A new keto-alcohol, (−)-mintlactone, (+)-isomintlactone and minor components in peppermint oil. Agr Biol Chem 1980;44: 1535–1543.

[2] Guth H. Determination of the configuration of wine lactone. Helv Chim Acta 1996;79: 1559–1570.

[3] Naf R, Velluz A. Phenols and lactones in Italo-Mitcham peppermint oil *Mentha* x *piperita* L. Flavour Fragance J 1998;13: 203–208.

[4] Gaudin JM. Synthesis and organoleptic properties of *p*-menthane lactones. Tetrahedron 2000;56: 4769–4776.

[5] Frerot S, Bagnound A, Vuilleumier C. Menthofurolactone: a new *p*-menthane lactone in *Mentha piperita* L.: analysis, synthesis and olfactory properties. Flavour Fragance J 2002;17: 218–226.

[6] Yukawa C, Imayoshi Y, Iwabuchi H, Komemushi S, Sawabe A. Chemical composition of three extracts of *Bursera graveolens* . Flavour Fragance J 2006;21: 234–238.

[7] Bourgeois MJ, Montaudon E. Base-induced reactions of *p*-menthane and pinane-derived epoxyesters—coconut fragrance. Helv Chim Acta 2001;84: 2430–2438.

[8] Dams I, Białońska A, Ciunik Z, Wawrzeńczyk C. Lactones 38: Synthesis and odoriferous properties of *p*-menthane lactones. Flavour Fragance J 2012;27: 237–243.

[9] Grudniewska A, Wawrzeńczyk C. Lactones 41. Synthesis and microbial hydroxylation of unsaturated terpenoid lactones with *p*-menthane ring systems. Molecules 2013;18: 2778–2787. 10.3390/molecules18032778 23455669 PMC6269982

[10] Wawrzeńczyk C, Dams I, Szumny A, Szczepanik M, Nawrot J, Prądzyńska A, et al Synthesis and evaluation of antifeedant, antifungal and antibacterial activity of isoprenoid lactones. Pol J Environ Stud 2005;14, Suppl. II: 69–84.

[11] Paruch E, Ciunik Z, Nawrot J, Wawrzeńczyk C. Lactones.9. Synthesis of terpenoid lactones—active insect antifeedants. J Agric Food Chem 2000;48: 4973–4977. 11052764 10.1021/jf991307o

[12] Dancewicz K, Gabryś B, Dams I, Wawrzeńczyk C. Enantiospecific effect of pulegone and pulegone-derived lactones on *Myzus persicae* (Sulz.) settling and feeding. J Chem Ecol 2008;34: 530–538. 10.1007/s10886-008-9448-9 18340487

[13] Grudniewska A, Dancewicz K, Białońska A, Ciunik Z, Gabryś B, Wawrzeńczyk C. Synthesis of piperitone-derived halogenated lactones and their effect on aphid probing, feeding, and settling behavior. RSC Adv 2011;1: 498–510.

[14] Grudniewska A, Dancewicz K, Białońska A, Wawrzeńczyk C, Gabryś B. Piperitone-derived saturated lactones: synthesis and aphid behavior-modifying activity. J Agric Food Chem 2013;61: 3364–3372. 10.1021/jf3052219 23477664

[15] Szczepanik M, Grudniewska A, Zawitowska B, Wawrzeńczyk C. Structure-related antifeedant activity of halolactones with a *p*-menthane system against the lesser mealworm, *Alphitobius diaperinus* Panzer. Pest Manag Sci 2014;70: 953–958. 10.1002/ps.3634 24009153

[16] Paruch E, Nawrot J, Wawrzeńczyk C. Lactones: Part 11. Feeding-deterrent activity of some bi- and tricyclic terpenoid lactones. Pest Manag Sci 2001;57: 776–780. 11561401 10.1002/ps.353

[17] Harmata J, Nawrot J. Comparison of the feeding deterent activity of some sesquiterpene lactones and lignan lactone towards selected insect storage pests. Biochem Syst Ecol 1984;12: 95–98.

[18] Nawrot J, Drożdż B, Holub M. Feeding deterrent activity of some natural sesquiterpene lactones for selected storage pests. Herba Pol 1985;XXXI: 209–212.

[19] Cis J, Nowak G, Kisiel W. Antifeedant properties and chemotaxonomic implications of sesquiterpene lactones and syringin from *Rhaponticum pulchrum* . Biochem Syst Ecol 2006;34: 862–867.

[20] Sosa ME, Tonn CE, Giordano OS. Insect antifeedant activity of clerodane diterpenoids. J Nat Prod 1994;57: 1262–1265. 7798961 10.1021/np50111a012

[21] Paruch E, Ciunik Z, Wawrzeńczyk C. Synthesis of spirolactones from the limonene system. Eur J Org Chem 1998;1998: 2677–2682.

[22] Dams I, Białońska A, Ciunik Z, Wawrzeńczyk C. Synthesis of terpenoid lactones with the *p*-menthane system. Eur J Org Chem 2004: 2662–2668.

[23] Goodwin MA, Waltman WD. Transmission of eimeria, viruses, and bacteria to chicks: darkling beetles (*Alphitobius diaperinus*) as vectors of pathogens. J Appl Poult Res 1996;5: 51–55. 10.1093/japr/5.1.51 PMC7129646 32288461

[24] Roche AJ, Cox NA, Richardson LJ, Buhr RJ, Cason JA, Fairchild BD, et al Transmission of *Salmonella* to broilers by contaminated larval and adult lesser mealworms, *Alphitobius diaperinus* (Coleoptera: Tenebrionidae). Poult Sci 2009;88: 44–48. 10.3382/ps.2008-00235 19096055

[25] Mota-Sanchez D, Hollingworth RM, Grafius EJ, Moyer DD. Resistance and cross-resistance to neonicotinoid insecticides and spinosad in the Colorado potato beetle, *Leptinotarsa decemlineata* (Say) (Coleoptera: Chrysomelidae). Pest Manag Sci 2006;62: 30–37. 16206238 10.1002/ps.1120

[26] Alyokhin A, Baker M, Mota-Sanchez D, Dively G, Grafius E. Colorado potato beetle resistance to insecticides. Am J Potato Res 2008;85: 395–413.

[27] Brault V, Uzest M, Monsion B, Jacquot E, Blanc S. Aphids as transport devices for plant viruses. C R Biol 2010;333: 524–538. 10.1016/j.crvi.2010.04.001 20541164

[28] Wińska K, Grudniewska A, Chojnacka A, Bialońska A, Wawrzeńczyk C. Enzymatic resolution of racemic secondary cyclic allylic alcohols. Tetrahedron: Asymmetry 2010;21: 670–678.

[29] Szczepanik M, Dams I, Wawrzeńczyk C. Feeding deterrent activity of terpenoid lactones with the *p*-menthane system against the Colorado potato beetle (Coleoptera: Chrysomelidae). Environ Entomol 2005;34: 1433–1440.

[30] Szczepanik M, Dams I, Wawrzeńczyk C. Terpenoid lactones with the *p*-menthane system as feeding deterrents to the lesser mealworm, *Alphitobius diaperinus* . Entomol Exp Appl 2008;128: 337–345.

[31] Rice SJ, Lambkin TA. A new culture method for lesser mealworm, *Alphitobius diaperinus* . J Appl Entomol 2009;133: 67–72.

[32] Rasband WS. ImageJ, U. S. National Institutes of Health. Bethesda, MD, USA 1997–2014. Available: http://rsb.info.nih.gov.

[33] Harrewijn P. Resistance mechanisms of plant genotypes to various aphid species In: Campbell RK, Eikenbary RD, editors. Aphid—plant genotype interactions. Amsterdam: Elsevier; 1990 pp. 117–130.

[34] Hardie J, Holyoak M, Taylor NJ, Griffiths DC. The combination of electronic monitoring and video-assisted observations of plant penetration by aphids and behavioural effects of polygodial. Entomol Exp Appl 1992;62: 233–239.

[35] Prado E, Tjallingii WF. Aphid activities during sieve element punctures. Entomol Exp Appl 1994;72: 157–165.

[36] Nawrot J, Błoszyk E, Harmatha J, Novotny L, Drożdż B. Action of antifeedants of plant origin on beetles infesting stored products. Acta Entomol Bohemos 1986;83: 327–335.

[37] Koul O. Bioassays In: Koul O, editor. Insect antifeedants. London: CRC Press; 2004 pp. 25–42.

[38] Hammer Ř, Harper DAT, Ryan PD. PAST: Paleontological Statistics Software Package for Education and Data Analysis. Palaeontol Electronica. 2001;4: 1–9. Available: http://palaeo-electronica.org/2001_1/past/issue1_01.htm.

[39] Tjallingii WF. Regulation of phloem sap feeding by aphids In: Chapman RF, de Boer G, editors. Regulatory mechanisms in insect feeding. New York: Chapman and Hall; 1995 pp. 190–209.

[40] López-Olguín J, de la Torre MC, Ortego F, Castañera P, Rodríguez B. Structure–activity relationships of natural and synthetic *neo*-clerodane diterpenes from *Teucrium* against Colorado potato beetle larvae. Phytochem 1999;50: 749–753.

[41] Tjallingii WF, Esch TH. Fine structure of aphid stylet routes in plant tissues in correlation with EPG signals. Physiol Entomol 1993;18: 317–328.

[42] Pettersson J, Tjallingii WF, Hardie J. Host-plant selection and feeding In: van Emden HF, Harrington R, editors. Aphids as crop pests. Wallingford: CABI; 2007 pp. 87–113.

[43] Mayoral AM, Tjallingii WF, Castañera P. Probing behaviour of *Diuraphis noxia* on five cereal species with different hydroxamic acid levels. Entomol Exp Appl 1996;78: 341–348.

[44] Frazier JL, Chyb S. Use of feeding inhibitors in insect control In: Chapman RF, de Boer G, editors. Regulatory mechanisms in insect feeding. New York: Chapman and Hall; 1995 pp. 364–381.

[45] Swain T. Secondary compounds as protective agents. Annu Rev Plant Physiol 1977;28: 479–501.

