# Supplementary figures and images for: Synthesis and Antifeedant Activity of Racemic and Optically Active Hydroxy Lactones with the p-Menthane System
Source: PLoS One. 2015 Jul 1;10(7):e0131028. doi: 10.1371/journal.pone.0131028 (PMC4488555; doi:10.1371/journal.pone.0131028)

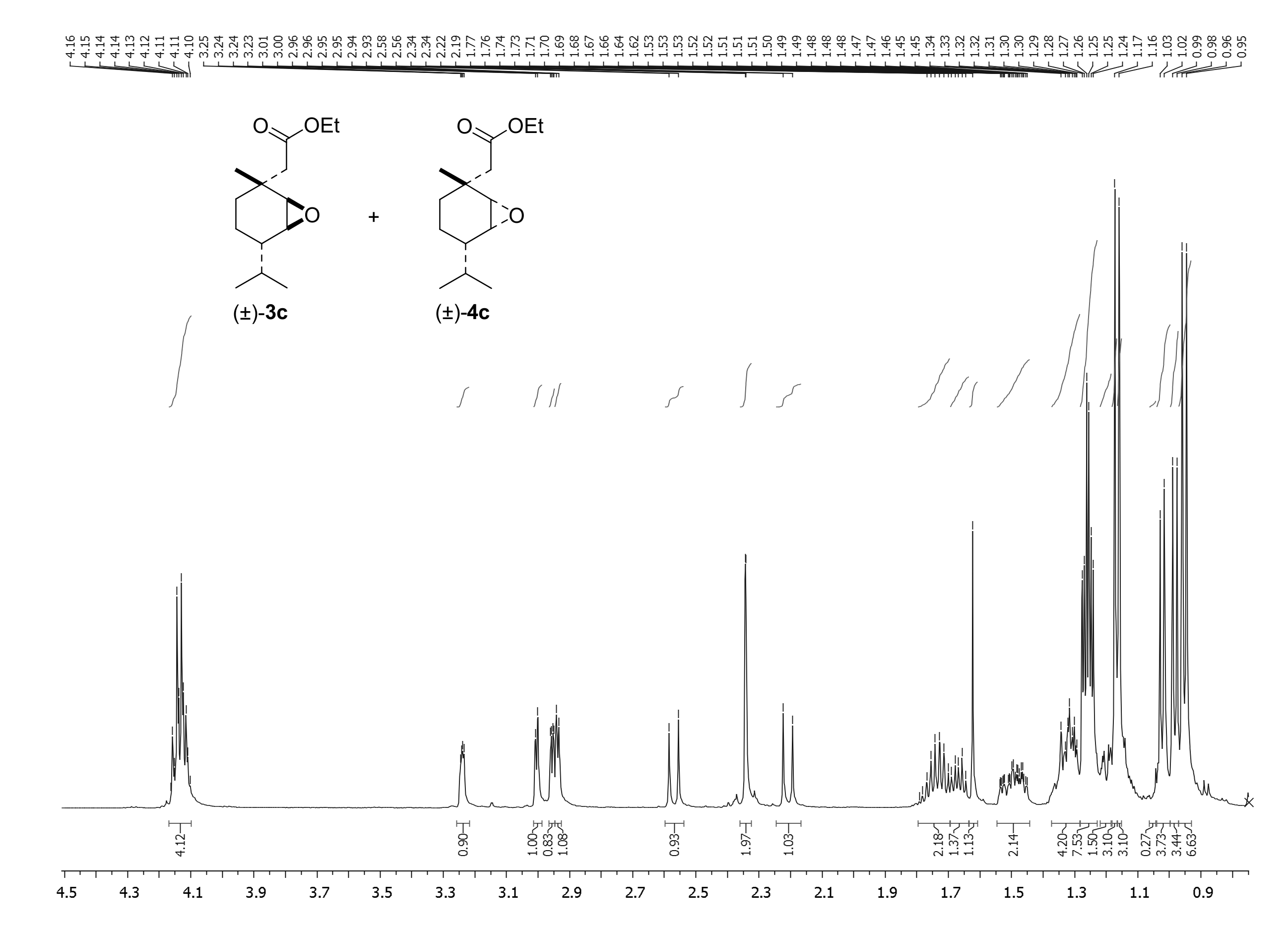

Supplement: S1 Fig — CDCl3, 300 MHz. (TIF) [file pone.0131028.s001.tif]

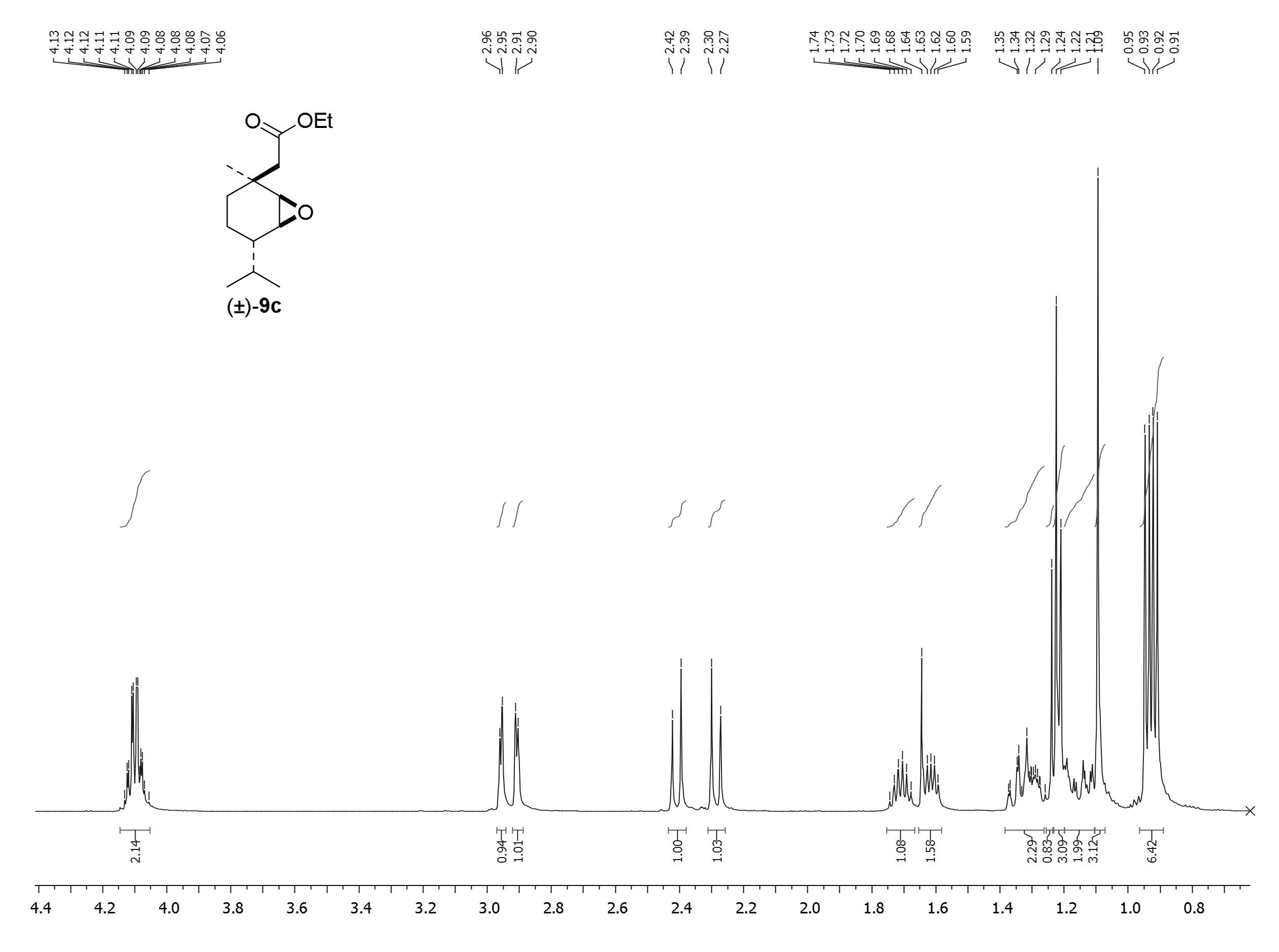

Supplement: S2 Fig — CDCl3, 300 MHz. (TIF) [file pone.0131028.s002.tif]

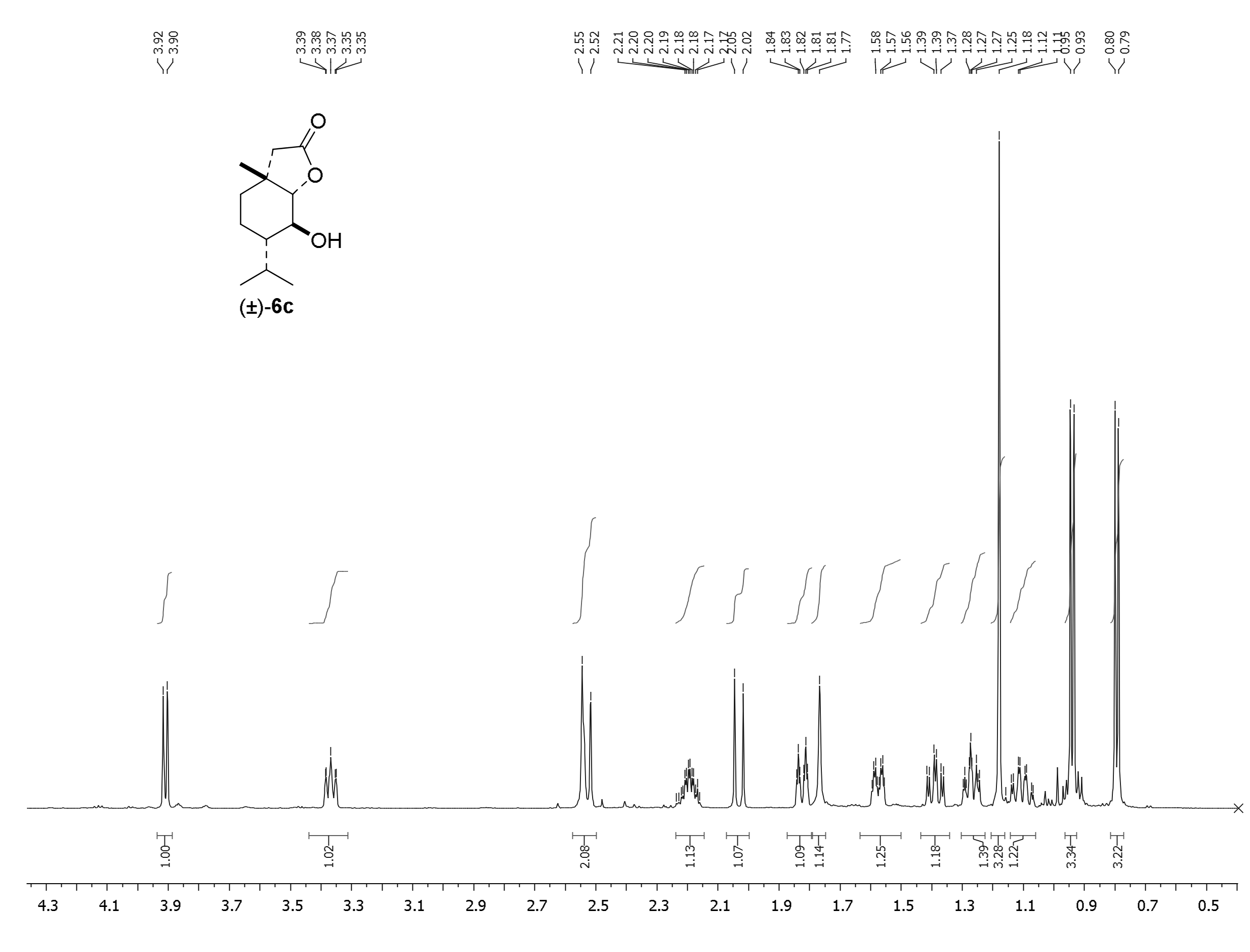

Supplement: S3 Fig — CDCl3, 600 MHz. (TIF) [file pone.0131028.s003.tif]

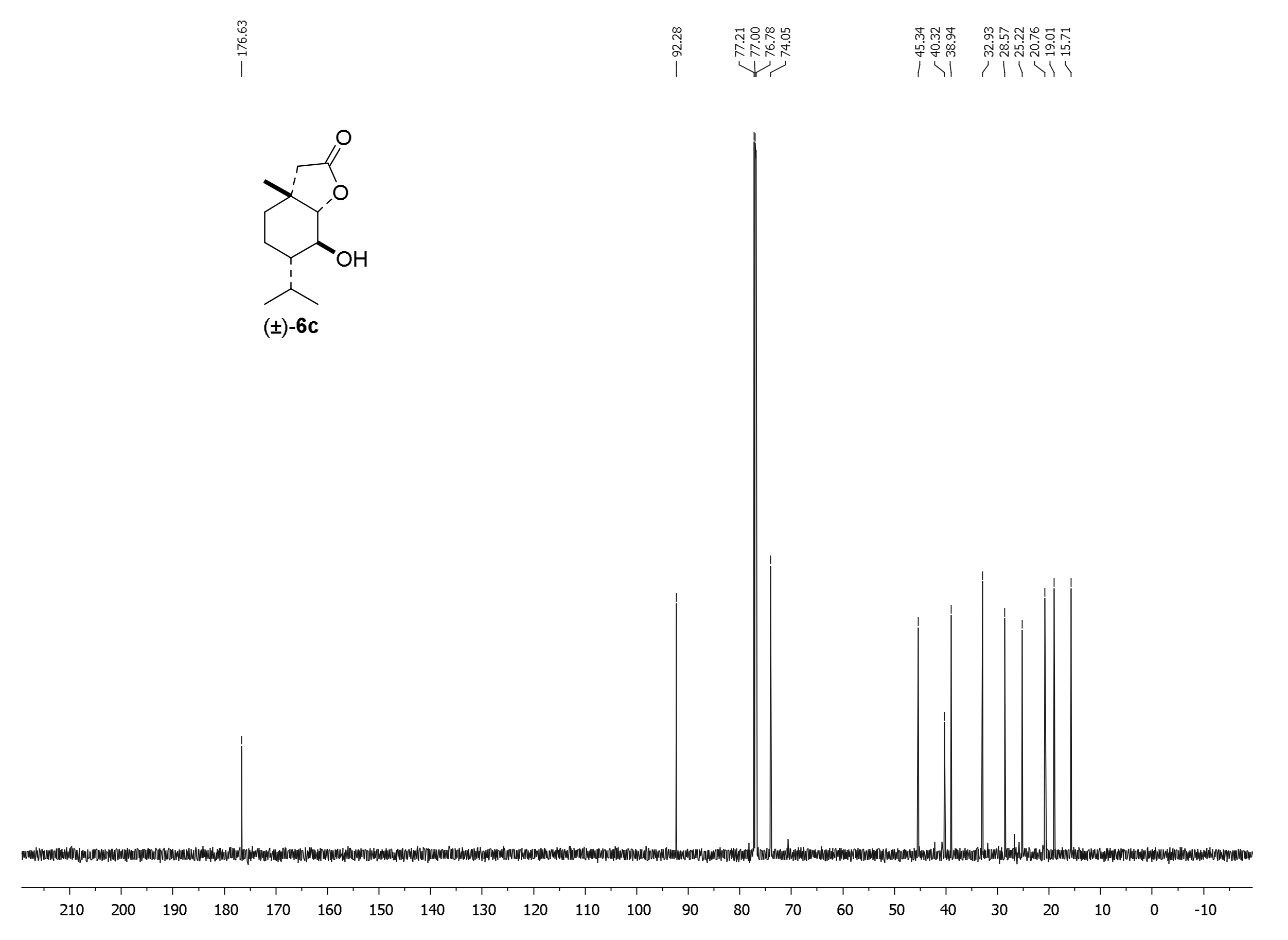

Supplement: S4 Fig — CDCl3, 151 MHz. (TIF) [file pone.0131028.s004.tif]

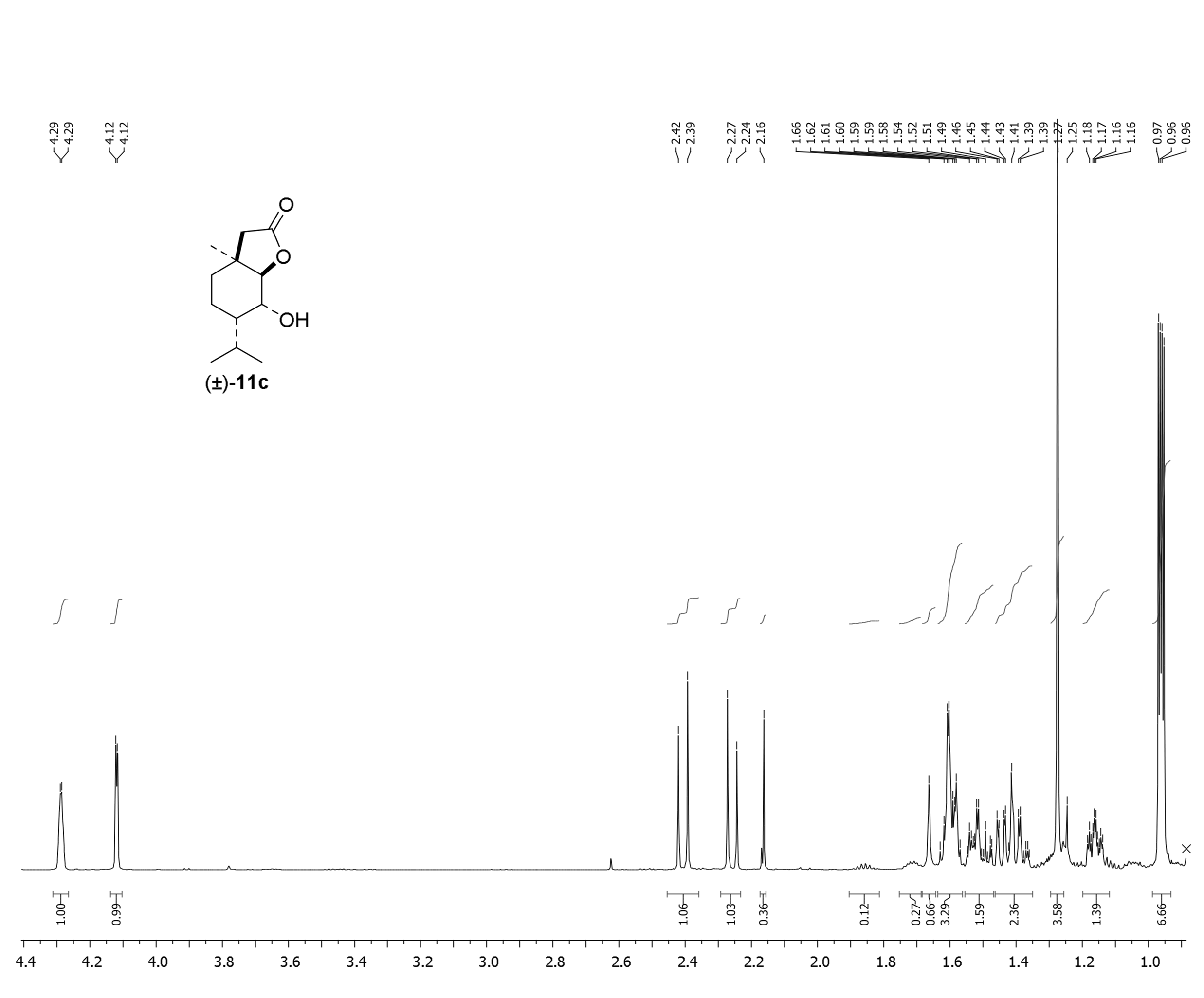

Supplement: S5 Fig — CDCl3, 600 MHz. (TIF) [file pone.0131028.s005.tif]

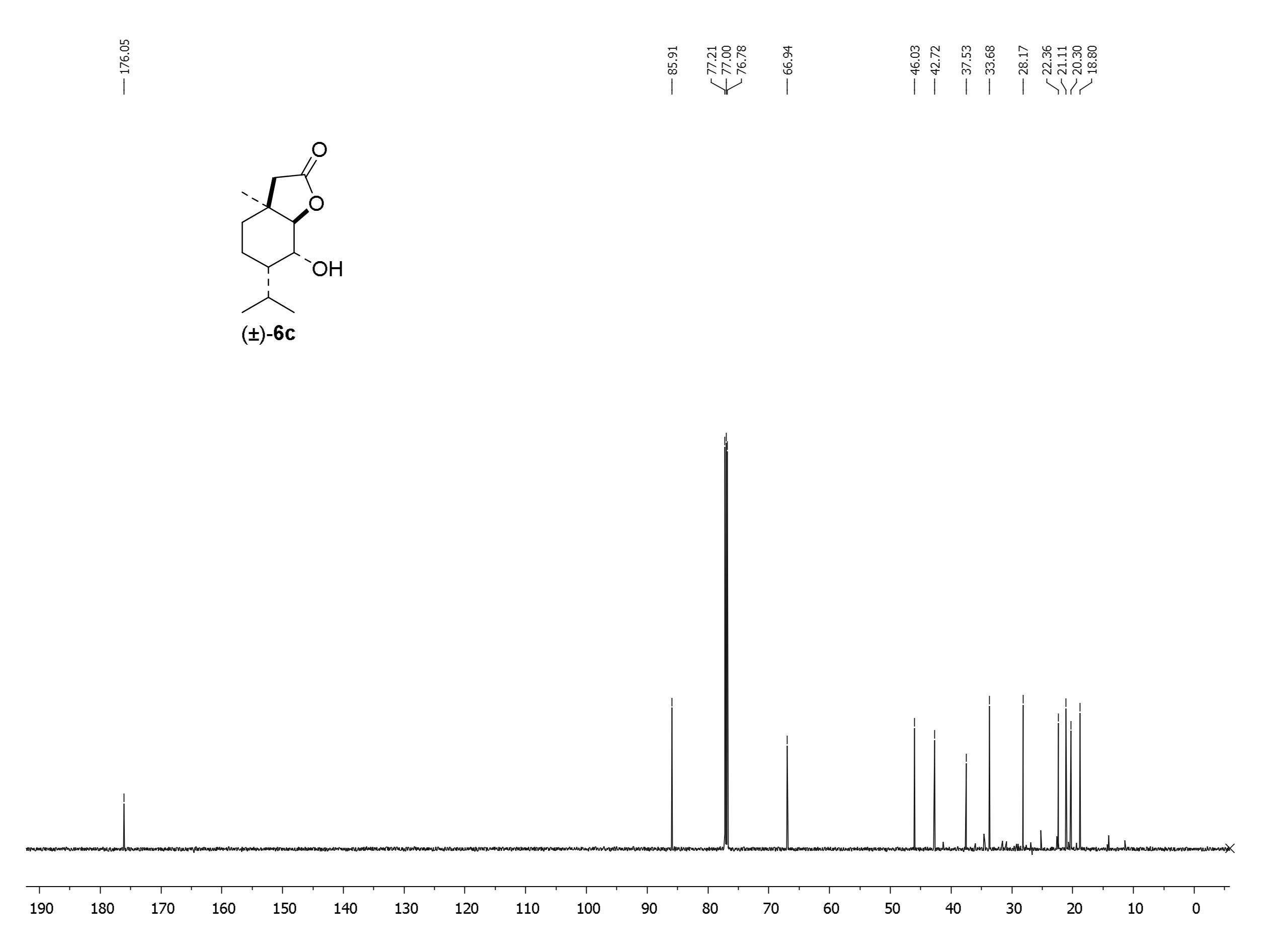

Supplement: S6 Fig — CDCl3, 151 MHz. (TIF) [file pone.0131028.s006.tif]

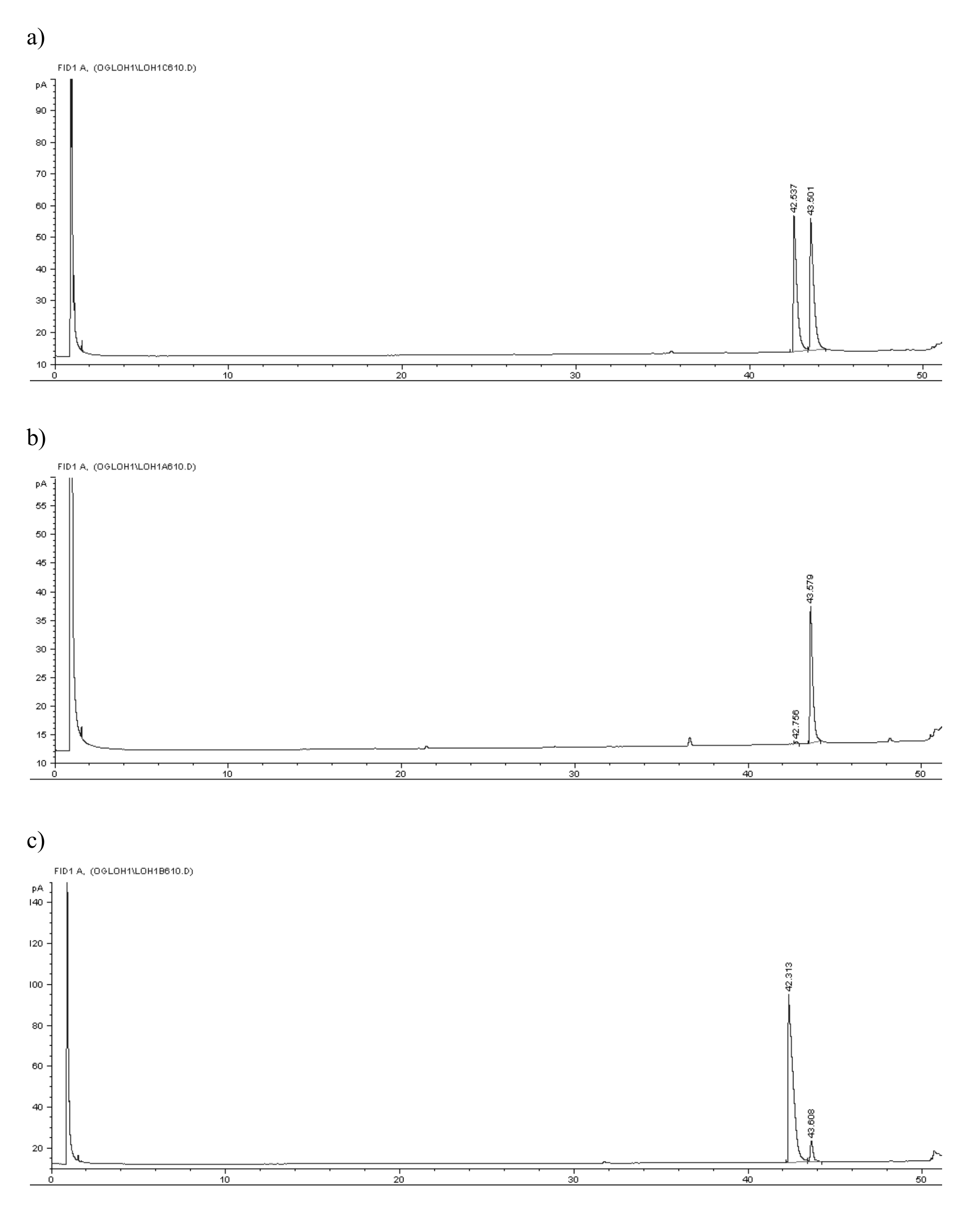

Supplement: S7 Fig — (a) (±)-6c; (b) (+)-6a, ee = 98%; (c) (−)-6b, ee = 91%. (TIF) [file pone.0131028.s007.tif]

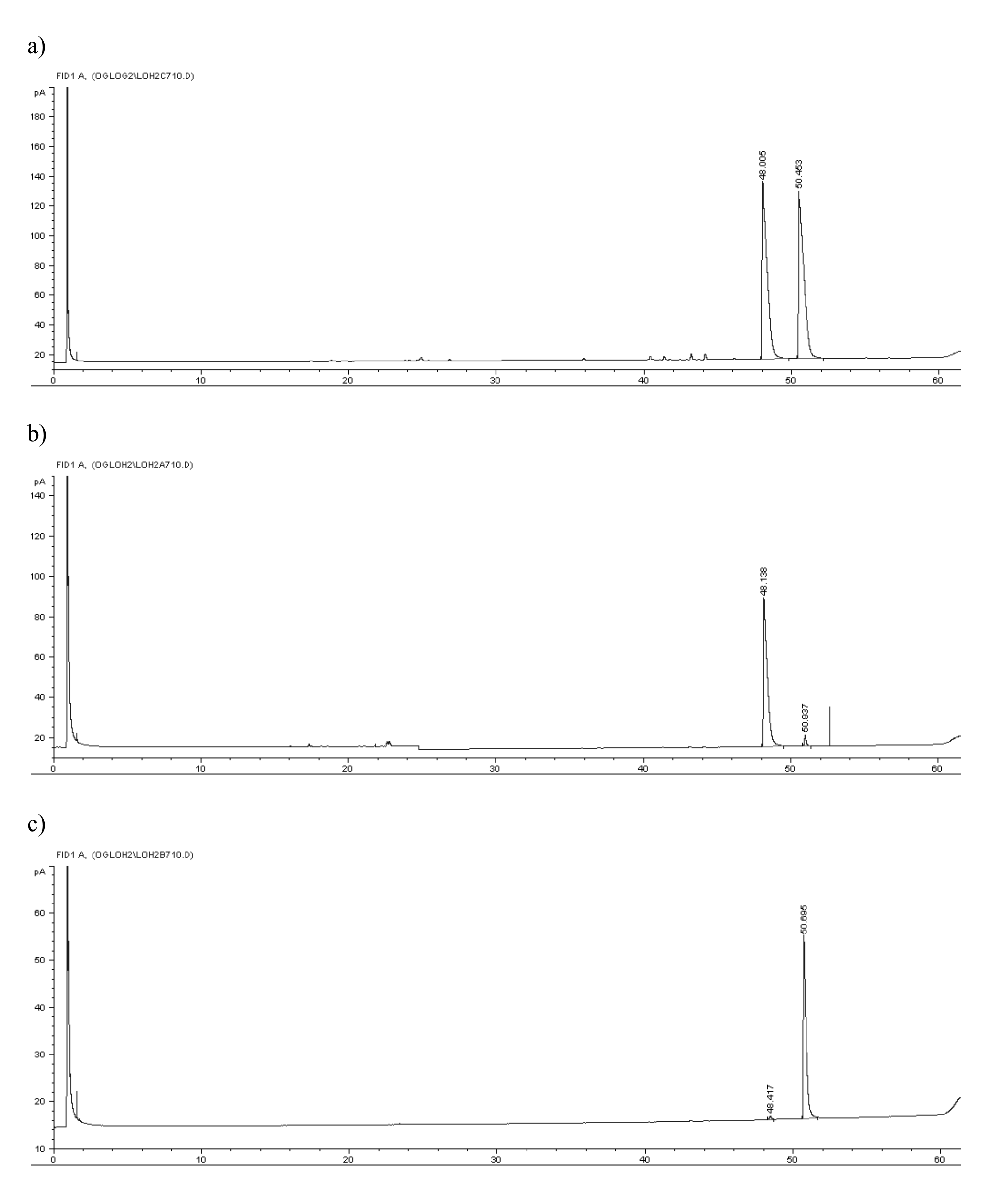

Supplement: S8 Fig — (a) (±)-11c; (b) (−)-11a, ee = 94%; (c) (+)-11b, ee = 98%. (TIF) [file pone.0131028.s008.tif]

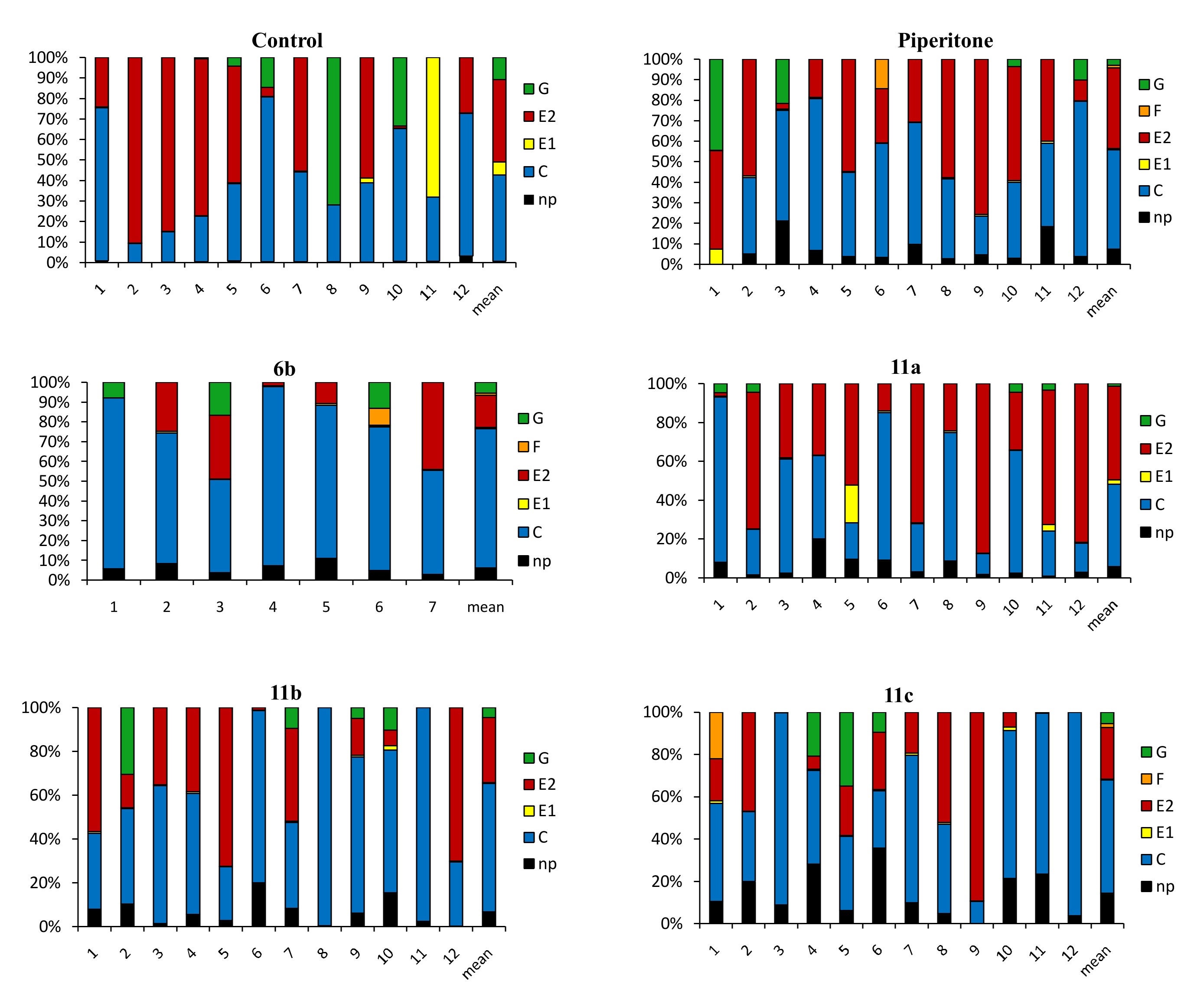

Supplement: S9 Fig — Numbers on the x-axis represent individual aphids. np, no probing; C, probing in parenchymatous tissues; E1, salivation in phloem; E2, ingestion of phloem sap; F, derailed stylet activities; G, ingestion of xylem sap (n = 12). (TIF) [file pone.0131028.s009.tif]

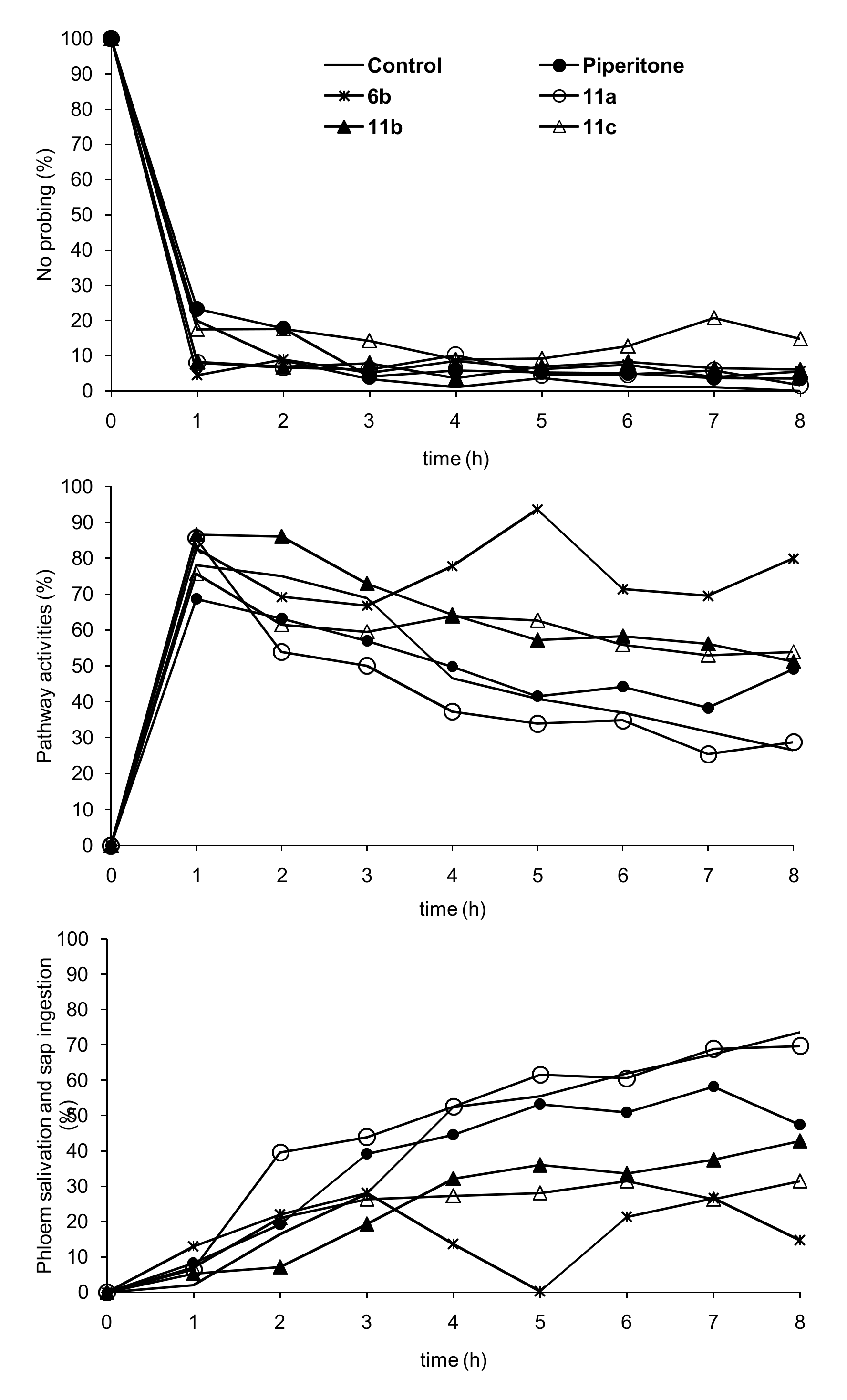

Supplement: S10 Fig — (TIF) [file pone.0131028.s010.tif]

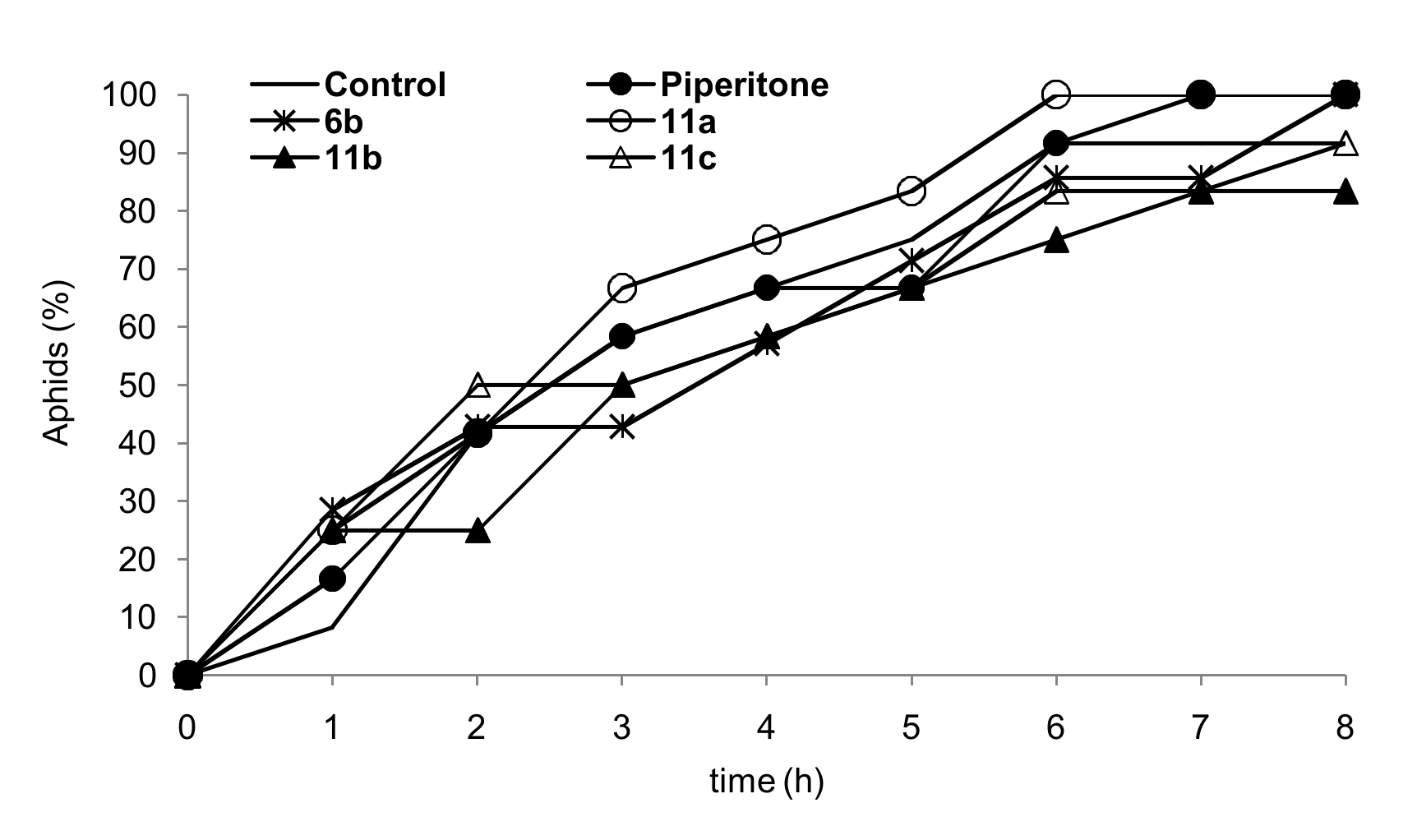

Supplement: S11 Fig — (TIF) [file pone.0131028.s011.tif]
