# Supplementary material for: Synthesis and Antifeedant Activity of Racemic and Optically Active Hydroxy Lactones with the p-Menthane System
Source: PLoS One. 2015 Jul 1;10(7):e0131028. doi: 10.1371/journal.pone.0131028 (PMC4488555; doi:10.1371/journal.pone.0131028)
Supplement: S1 File — Insect culture and feeding deterrent activity tests. (DOCX) [file pone.0131028.s012.docx]

S1 File

BIOASSAYS

**Insect Culture**

Experiments were conducted using the lesser mealworm, *Alphitobius diaperinus* (Panzer); Colorado potato beetle, *Leptinotarsa decemlineata* (Say); and peach-potato aphid, *Myzus persicae* (Sulz.).

A laboratory-reared strain of the *A*. *diaperinus* collected from a broiler farm located near Toruń, Poland (53^○^01′N, 18^○^37′E) was used in this study. The colony was kept in glass containers in a rearing chamber at +29^○^C in the dark. A mixture consisting of one part oat flakes, one part wheat bran, and 0.01 part brewer’s yeast was provided as food. To maintain moisture levels at ca. 55%, fresh apple halves were placed in the containers. Larvae (25−30 days old) and unsexed adults (7−10 days old) of *A. diaperinus* were used for the experiments. To obtain large numbers of *A*. *diaperinus* of approximately the same age, the Rice–Lambkin culture method was used [S[1](#_ENREF_1)].

The first generation of *L. decemlineata* adults was collected from an unsprayed potato field. Eggs were collected and hatched in the laboratory. Larvae were reared on potato leaves in Petri dishes at 24°C and 60% relative humidity in a climate chamber, with a 16:8 h light-dark photoperiod.

Laboratory clones of *M*. *persicae* were maintained on Chinese cabbage, *Brassica pekinensis* in the laboratory at 20^○^C and 65% relative humidity, with a 16:8 h light-dark photoperiod. Apterous females of *M. persicae* (1−7 days old) and 3-week-old plants with 4−5 fully developed leaves were used for the experiments.

**Feeding Deterrent Activity Tests**

Choice and no-choice tests were used according to the procedures described previously [S[2-S4](#_ENREF_2)]. The choice test was highly sensitive, but insects could easily avoid treated food. The no-choice test design most closely approaches a practical application.

***Chewing Insects (A. diaperinus and L. decemlineata)***

In the experiments with *A*. *diaperinus,* oat flakes purchased from Melvit S.A. (Warsaw, Poland) were used as the test food. For the feeding assays, 1% acetone solutions of the test compounds were prepared. A dose (1 mL) of the solution or acetone alone as control was applied to the flakes (1g) by using a micropipette. After evaporation of the solvent in air for 30 min, the flakes were weighed and offered to 10 larvae or unsexed adults during the following 3-day period. In the choice test, both control and treated oat flakes were placed together in a Petri dish (15-cm diameter), with the control flakes separated from the treated flakes by using a thin glass capillary. In the no-choice test, insects were exposed to only one kind of food-treated or control. Dishes were maintained in the rearing chamber at 29 ± 1°C in the dark.

The experiments with *L. decemlineata* were conducted according to the classical leaf-disk bioassay described earlier [S[2](#_ENREF_2)], by using adults collected every day from an unsprayed potato field and newly molted third-instar larvae obtained from a laboratory colony. For the feeding tests, 1% ethanol solutions of the compounds were used. By using a cork borer (4 cm diameter), disks were cut from potato leaves immediately before the application of treatment solutions. After the leaf disks were cut, they were immediately dipped in the test solutions or alcohol as the control. After the solvent had completely evaporated, two each of the solvent control and treated disks were placed on top of moist filter paper in Petri dishes (15 cm diameter) along with 6 adults (3 pairs) or 10 larvae (choice test). In the no-choice test, only control or only treated disks were placed in the dishes. In each of the four replicates, the insects were allowed to feed *ad libitum* for 24 h at 24°C under a 16:8 h light-dark photoperiod. After the completion of the experiments, the oat flakes were reweighed (*A*. *diaperinus*), and the areas of the remaining uneaten potato leaf disks (*L. decemlineata*) were measured using a scanner and ImageJ image analysis software [S[5](#_ENREF_5)]. From the test data for *A. diaperinus* and *L*. *decemlineata*, the deterrence coefficients (relative *R*, and absolute *A*) were calculated (Equations 1 and 2).

***Aphids (M. persicae)***

In the experiment with *M. persicae,* the settling choice-test was applied, as described previously [S[4](#_ENREF_4)]. Aphids settle on a plant only when they accept it as a food source [S[6](#_ENREF_6)]. Therefore, the number of aphids that settle and feed on a given substrate is a good indicator of its suitability. This bioassay allowed us to study aphid host preferences under seminatural conditions. Aphids were given free choice between control and treated leaves. The studied lactones were applied by immersing a leaf in a 0.1% ethanolic solution of a given compound for 30 s. Control leaves of similar size were immersed in 70% ethanol that was used as the solvent for the studied lactones. In this bioassay, treated and control leaves were placed in a Petri dish and allowed to dry for 1 h to permit evaporation of the solvent. Subsequently, the aphids were placed in the dish along a line that divided the area into two halves, so that the aphids could choose between treated (on one-half of the Petri dish) and control leaves (on the other half of the dish). Aphids that settled (i.e. they did not move) and indicated feeding based on the position of their antennae [S[7](#_ENREF_7)]were counted on each leaf at 1, 2, and 24-h intervals (8 replicates, 20 viviparous apterous females/replicate). Aphids that were moving or away from any of the leaves were not counted. The test data were analyzed using the Student’s *t*-test. If the aphids showed a clear preference for the leaf treated with the studied compound (*p* < 0.05), the compound was described as having attractant properties. If the aphids settled mainly on the control leaf (*p* < 0.05), the compound studied in the respective choice test was designated a deterrent. Thus, from the data obtained, the relative index of deterrence (*DI*) was calculated (Equation 5).

In the experimental setup for the electrical penetration graph (EPG), the aphid and plant comprise parts of an electric circuit that is completed when the aphid inserts its stylets into the plant. A weak voltage is supplied to the circuit, and all changing electric properties are recorded as EPG waveforms that can be correlated with aphid activities and stylet position in plant tissues [S[8](#_ENREF_8)]. The lactones were applied to a 3-week-old plant by immersing one leaf in a 0.1% ethanolic solution of a given compound for 30 s. Control leaves of similar size were immersed in the 70% ethanol that was used as the solvent for the tested lactones. The treated and control leaves were allowed to dry for 1 h before the start of the experiment to permit the evaporation of the solvent. Aphids were attached to a Au wire electrode with conductive silver paint and starved for 1 h prior to the experiment. The probing behaviors of 12 apterous females per studied lactone/aphid combination were monitored for 8 h continuously with four-channel DC EPG recording equipment. Each aphid was given access to a freshly prepared leaf. Signals were saved on the computer and analyzed using the PROBE 3.1 software provided by Dr. W. F. Tjallingii (EPG-Systems, Dillenburg 12, 6703 CJ Wageningen, The Netherlands; www.epgsystems.eu). The following aphid behaviors were distinguished: no penetration (waveform np, aphid stylets outside the plant); pathway phase, penetration of nonphloem tissues (waveforms ABC); salivation into sieve elements (waveform E1); ingestion of phloem sap (waveform E2); and ingestion of xylem sap (waveform G). The results were statistically analyzed using the Mann−Whitney *U* test at *p* < 0.05.

**References (S1 File)**

S1. Rice SJ, Lambkin TA. A new culture method for lesser mealworm, *Alphitobius diaperinus*. J Appl Entomol. 2009;133: 67-72.

S2. Szczepanik M, Dams I, Wawrzeńczyk C. Feeding deterrent activity of terpenoid lactones with the *p*-menthane system against the Colorado potato beetle (Coleoptera: Chrysomelidae). Environ Entomol. 2005;34: 1433-1440.

S3. Szczepanik M, Dams I, Wawrzeńczyk C. Terpenoid lactones with the *p*-menthane system as feeding deterrents to the lesser mealworm, *Alphitobius diaperinus*. Entomol Exp Appl. 2008;128: 337-345.

S4. Grudniewska A, Dancewicz K, Białońska A, Ciunik Z, Gabryś B, Wawrzeńczyk C. Synthesis of piperitone-derived halogenated lactones and their effect on aphid probing, feeding, and settling behavior. RSC Adv. 2011;1: 498-510.

S5. Rasband WS. ImageJ, U. S. National Institutes of Health. Bethesda, MD, USA 1997-2014. Available: <http://rsb.info.nih.gov/19/2/14>

S6. Harrewijn P. Resistance mechanisms of plant genotypes to various aphid species. In: Campbell RK, Eikenbary RD, editors. Aphid - plant genotype interactions. Amsterdam: Elsevier; 1990. pp. 117-130.

S7. Hardie J, Holyoak M, Taylor NJ, Griffiths DC. The combination of electronic monitoring and video-assisted observations of plant penetration by aphids and behavioural effects of polygodial. Entomol Exp Appl. 1992;62: 233-239.

S8. Prado E, Tjallingii WF. Aphid activities during sieve element punctures. Entomol Exp Appl. 1994;72: 157-165.
