# Supplementary material for: Synthesis and Antifeedant Activity of Racemic and Optically Active Hydroxy Lactones with the p-Menthane System
Source: PLoS One. 2015 Jul 1;10(7):e0131028. doi: 10.1371/journal.pone.0131028 (PMC4488555; doi:10.1371/journal.pone.0131028)
Supplement: S2 File — (DOCX) [file pone.0131028.s013.docx]

S2 File

Aphid probing

The EPG recordings revealed all aphid activities related to plant penetration: nonprobing (np) and pathway phase (C), including unidentified (derailed) stylet movements (F), phloem salivation (E1), phloem sap ingestion (E2), and xylem sap uptake (G). Activities F and G occurred sporadically irrespective of the treatment. Despite high individual variation among the aphids within the experimental groups (S9 Fig.), certain trends in aphid behavior could be observed. The typical behavior on the control untreated plants included a short total time for np activities, i.e., the period when aphid stylets were removed from plant tissues. This comprised 5% of the experimental time, and the breaks between probes were short, 3 min on average. The remaining time of the 8 h EPG recording was equally divided into pathway and phloem activities. The individual probes were relatively long (2.5 h average duration), and the number of failed probes before finding phloem was low (6 per aphid), despite the prolonged total time to the first sap ingestion period E2 (2.6 h). However, the mean duration of pathway before the first phloem phase within a successful probe was relatively short (ca. 40 min). The individual period of phloem sap ingestion lasted 3 h on average (Table 3). In 4 h from the onset of the experiment, 50% of the aphids reached sieve elements and the overall success rate in the 8 h experiment was 92% (S9-S11 Figs). Piperitone did not evoke significant differences in *Myzus persicae* probing behavior in comparison to the control. The duration of probing and the proportion of phloem phase in total probing were similar in aphids on control and piperitone-treated leaves. All aphids reached phloem vessels and most of them showed sustained phloem sap ingestion during the first probe. Interestingly, the proportion of salivation into sieve elements during the phloem phase was five times lower for aphids on the treated leaves than on the control leaves. The prolonged phloem salivation is a likely indication of the aphid response to negative chemical factors in the plant sap. In consequence, aphids were clearly attracted to settle on piperitone-treated leaves 24 h after application (Fig. 3 and S9-S11 Figs). The application of the hydroxy lactones had varying effects on aphid behavior. Lactones **11a** and **11b** did not cause any changes in the probing behavior during the 8 h after application, which was the duration of the EPG experiment: the values of probing-related EPG parameters were similar to those in aphids on the control and piperitone-treated plants. In contrast, the application of **6b** and **11c** did modify aphid responses to the host-plants. On **6b**-treated plants, the total duration of probing was similar to that on the control plants, but probing comprised mainly pathway activity: phloem sap ingestion contributed only 18% of probing time, in contrast to 46% on the control plants (Table 3). Aphids were not deterred from probing – the total number of probes was four times higher but the probes were seven times shorter than on the control plants. In relation to piperitone, these values were 1.4 and 1.7, respectively. Moreover, the number of short (< 3 min) epidermal probes was eight times higher than on the control plants. 85% of the aphids reached sieve elements, but only 67% of them showed sustained (i.e., longer than 10 min) phloem sap ingestion activity. At the same time, the average duration of sap ingestion periods (E2) was five times shorter. However, the times to reach phloem elements within a successful probe were 2.5 and 2.1 times shorter in aphids on **6b**-treated plants than on control and piperitone-treated, respectively. There were also 15- and 2-fold increases in the number of probes after the sap ingestion period, as compared to the control and piperitone, respectively (Table 3, S9-S11 Figs). On **11**-treated plants, the total durations of aphid non-probing activities were three and two times longer than on the control and piperitone-treated plants. However, within probing, there were no significant differences in the durations of pathway and phloem-related activities. 83% of the aphids reached sieve elements and started ingestion and, in 90%, the ingestion was sustained. However, the mean duration of sap ingestion was two times shorter than on the control plants, on average (Table 3, S9-S11 Figs).
